# Progressive alterations in amino acid and lipid metabolism correlate with peripheral neuropathy in *Polg*^D257A^ mice

**DOI:** 10.1126/sciadv.abj4077

**Published:** 2021-10-15

**Authors:** Esther W. Lim, Michal K. Handzlik, Elijah Trefts, Jivani M. Gengatharan, Carlos M. Pondevida, Reuben J. Shaw, Christian M. Metallo

**Affiliations:** 1 Department of Bioengineering, University of California San Diego, La Jolla, CA 92093, USA.; 2 Molecular and Cell Biology Laboratory, Salk Institute for Biological Studies, La Jolla, CA, 92037, USA.

## Abstract

Mitochondria are central to metabolic homeostasis, and progressive mitochondrial defects have diverse metabolic consequences that could drive distinct pathophysiological states. Here, we comprehensively characterized metabolic alterations in *Polg*^D257A^ mice. Plasma alanine increased markedly with time, with other organic acids accumulating to a lesser extent. These changes were reflective of increased Cori and Cahill cycling in *Polg*^D257A^ mice and subsequent hypoglycemia, which did not occur during normal mouse aging. Tracing with [^15^N]ammonium further supported this shift in amino acid metabolism with mild impairment of the urea cycle. We also measured alterations in the lipidome, observing a reduction in canonical lipids and accumulation of 1-deoxysphingolipids, which are synthesized from alanine via promiscuous serine palmitoyltransferase activity and correlate with peripheral neuropathy. Consistent with this metabolic link, *Polg*^D257A^ mice exhibited thermal hypoalgesia. These results highlight the longitudinal changes that occur in intermediary metabolism upon mitochondrial impairment and identify a contributing mechanism to mitochondria-associated neuropathy.

## Teaser

Alanine accumulation in *Polg*^D257A^ mice links mitochondrial defects to 1-deoxysphingolipids and peripheral neuropathy.

## INTRODUCTION

Mitochondria are multifaceted organelles that are critical for adenosine 5′-triphosphate (ATP) production, maintenance of redox homeostasis, biosynthesis of metabolites, and recycling of metabolic byproducts (*1*). Aberrations in mitochondrial DNA (mtDNA) arise through diverse mechanisms including reactive oxygen species (ROS)–mediated oxidative damage (*2*), impaired base excision repair (*3*), or decreased polymerase γ (POLG) fidelity (*4*). More generally, sustained, low-level mutational stress first affects mitochondrial function, as evidenced by the impacts of low-concentration ethidium bromide (*5*, *6*) on mitochondria. Such changes will manifest in diverse metabolic alterations as mitochondrial components become depleted across different tissues.

The *Polg*^D257A^ mtDNA mutator (*Polg*) mouse is a model of progressive mitochondrial dysfunction (*7*, *8*). *Polg* mice carry a missense mutation that significantly reduces 3′-5′ exonuclease activity required for proofreading, leading to a marked increase in mtDNA mutations and reduced expression of proteins involved in the electron transport chain (ETC) (*9*, *10*). These mice display progeroid phenotypes including alopecia, loss of body fat, sarcopenia, kyphosis, anemia, osteoporosis, reduced fertility, cardiomyopathy, and a shortened life span (*7*, *8*). Ultimately, this model results in severe mitochondrial functional deficiency that contributes to these diverse phenotypes (*7*, *11*, *12*). In humans, mutations in the *POLG* gene are associated with a spectrum of mitochondrial diseases including Alpers-Huttenlocher syndrome, progressive external ophthalmoplegia, myoclonic epilepsy myopathy sensory ataxia, ataxia neuropathy spectrum, and myocerebrohepatopathy spectrum disorders (*13*). The diagnosis of these mitochondrial diseases is often challenging due to the overlapping range of symptoms with multiple organs and a wide range of severity and timing of onset.

Mitochondrial respiration is inextricably tied to central carbon metabolism, and the progressive metabolic compensation that occurs in *Polg* mice as they accumulate and adapt to mtDNA mutations has not been explored in detail. By observing changes in metabolism that occur in *Polg* mice over time, we hope to identify key biochemical processes that could be exploited to mitigate phenotypes associated with mitochondrial dysfunction. Here, we comprehensively quantified the metabolic alterations and fluxes in *Polg* mice between the ages of 3 to 11 months and compared them to age-matched wild-type (WT) controls. Among the metabolites that differed between WT and *Polg* mice, alanine stood out with its levels increasing markedly over time as early as 4 months of age, even preceding changes in lactate. Using ^13^C and ^15^N dynamic labeling, we highlight specific changes in central carbon metabolism and nitrogen homeostasis indicative of defects in tricarboxylic acid (TCA) and urea cycles. In addition, we observed significant alterations in lipid metabolism downstream of these effects, including the accumulation of alanine-derived 1-deoxysphingolipids (doxSLs). Pathologically, doxSLs have been associated with hereditary sensory and autonomic neuropathy type 1 (HSAN1) (*14*), diabetic sensory neuropathy (*15*, *16*), and paclitaxel-induced neuropathy (*17*). Consistent with this metabolic alteration, we reveal the development of thermal hypoalgesia in *Polg* mice, mechanistically linking mitochondrial defects with peripheral neuropathy through amino acid and sphingolipid metabolism. The longitudinal metabolic data from *Polg* mice can serve as a roadmap for additional drivers of pathophysiologies associated with mitochondrial dysfunction.

## RESULTS

### Impaired bioenergetic metabolism

**Fig. 1.**
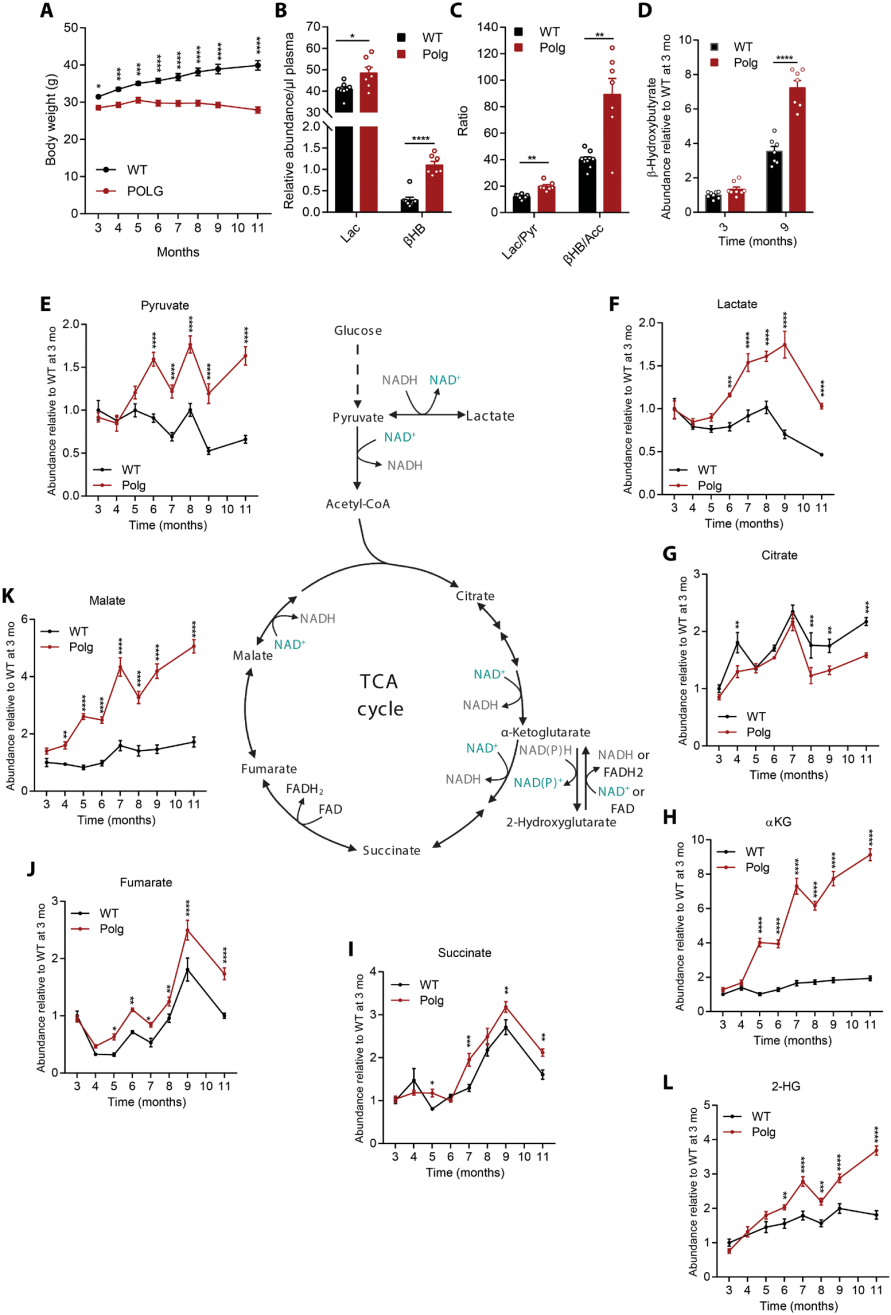
*Polg* mice have impaired bioenergetic metabolism. (**A**) Body weight of WT and *Polg* mice over time. (**B**) Abundance of lactate (Lac) and β-hydroxybutyrate (βHB) relative to internal standard per microliter of plasma in WT and *Polg* mice at 12 months of age. (**C**) Lactate:pyruvate (Lac/Pyr) and β-hydroxybutyrate:acetoacetate (βHB/Acc) ratio in WT and *Polg* plasma at 12 months of age. (**D**) Abundance of β-hydroxybutyrate relative to internal standard per microliter of plasma at 3 and 9 months of age. Abundance of plasma pyruvate (**E**), lactate (**F**), citrate (**G**), α-ketoglutarate (αKG) (**H**), succinate (**I**), fumarate (**J**), malate (**K**), and 2-hydroxyglutarate (2-HG) (our measurement does not distinguish between D and L enantiomers) (**L**) relative to internal standard, normalized to WT values at 3 months. Two-way analysis of variance (ANOVA) (A, D, and E to L) or two-sided Student’s *t* test (B and C) for each comparison with no adjustment for multiple comparisons. Data are means ± SEM of *n* = 7 to 8 animals per group. *P < 0.05, **P < 0.01, ***P < 0.001, and ****P < 0.0001.

We monitored *Polg* mouse body weight monthly from 3 to 11 months and observed minimal weight gain as they aged (Fig. 1A). These results not only are consistent with previously published *Polg* cohorts (*7*, *8*) but also suggest that substantial metabolic alterations are present at an early age. To assess progressive changes in metabolism, we analyzed plasma obtained from the tail vein from WT and *Polg* mice every month until they were sacrificed at 12 months old.

*Polg* mice should accumulate mitochondrial dysfunction as they age; hence, we first quantified common mitochondrial dysfunction markers in plasma and saw elevated levels of lactate and β-hydroxybutyrate (Fig. 1B). These metabolites are products of key oxidative substrates for tissues throughout the body and reflect alterations in the NAD^+^ (nicotinamide adenine dinucleotide):NADH (reduced form of NAD^+^) ratio. As expected, the lactate:pyruvate and β-hydroxybutyrate:acetoacetate ratios were significantly increased in *Polg* mice (Fig. 1C). Notably, levels of β-hydroxybutyrate were similar at 3 months of age but were significantly elevated in aged *Polg* mice (Fig. 1D). We next quantified organic acids in plasma collected monthly as the mice aged. WT and *Polg* mice had similar levels of pyruvate and lactate through 5 months of age, but levels of each increased substantially in *Polg* mice plasma from 6 months onward (Fig. 1, E and F).

The TCA cycle in the mitochondrial matrix consists of a series of redox reactions to harness energy in the forms of NADH, FADH_2_ (reduced flavin adenine dinucleotide), and ATP. The electrons in NADH and FADH_2_ are then funneled to the ETC through oxidative phosphorylation to generate more ATP. These pathways are coupled in most mitochondria, so accumulation of NADH in the context of reduced mitochondrial function will reduce oxidative TCA metabolism. Consistent with a potential decrease in pyruvate dehydrogenase (PDH) and TCA cycle flux, we observed a reduction in citrate in *Polg* mice plasma relative to WT mice (Fig. 1G). On the other hand, there was a substantial accumulation of TCA cycle intermediates such as α-ketoglutarate, succinate, fumarate, and malate in *Polg* mice compared to WT (Fig. 1, H to K). Notably, α-ketoglutarate and malate levels increased strikingly over time in *Polg* mice, indicating that TCA metabolites are differentially affected by progressive mtDNA accumulations. Succinate levels in aged *Polg* and WT mice differed by only 1.5-fold compared to ~3- or 5-fold for malate and α-ketoglutarate, respectively (Fig. 1, H, I, and K). Levels of 2-hydroxyglutarate (2-HG) were also elevated in *Polg* mouse plasma compared to WT (Fig. 1L), trending with α-ketoglutarate. Overall, the accumulation of these metabolites reveals a progressive impairment in bioenergetic metabolism and redox stress that becomes evident as early as 4 months of age for certain metabolites in *Polg* mice (Fig. 1, E to L).

### Differential amino acid metabolism

**Fig. 2.**
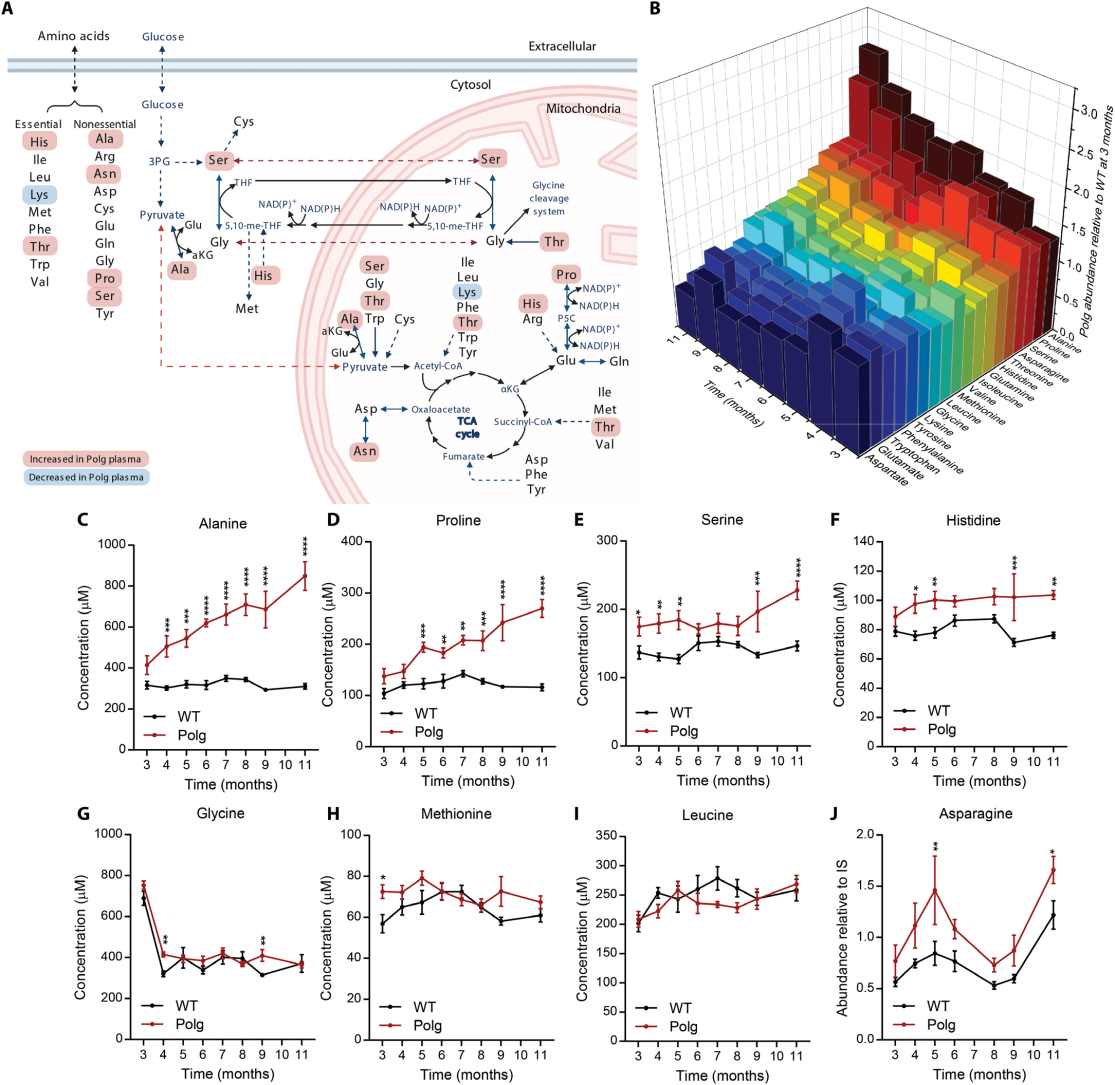
Alanine and proline accumulate in *Polg* mice plasma with age. (**A**) Simplified pathway map of mitochondrial amino acid metabolism. (**B**) Three-dimensional plot of average plasma amino acid fold change in *Polg* mice relative to WT mice at 3 months. Refer to table S1 for plasma amino acid concentration. Concentration of plasma alanine (**C**), proline (**D**), serine (**E**), histidine (**F**), glycine (**G**), methionine (**H**), and leucine (**I**) in WT and *Polg* mice from 3 to 11 months of age. (**J**) Abundance of plasma asparagine relative to internal standard (IS) from 3 to 11 months of age. Two-way ANOVA for each comparison with no adjustment for multiple comparisons. Data are means ± SEM of *n* = 7 to 8 animals per group. *P < 0.05, **P < 0.01, ***P < 0.001, and ****P < 0.0001.

Mitochondria and complex I of the ETC are intimately linked to amino acid metabolism given their role in α-keto acid oxidation (Fig. 2A). We observed significant alterations in several amino acids within *Polg* plasma, with some increasing further over time (Fig. 2B). Alanine increased steadily over time in *Polg* mice but remained unchanged in aging WT mice (Fig. 2C). This finding mirrors the observed increase in pyruvate and lactate (Fig. 1, E and F) as well as findings in mitochondrial disease patients (*18*). However, the rise in alanine preceded that of other metabolites such as pyruvate and lactate (Fig. 1, E and F) by 2 months, suggesting that alanine levels are extremely responsive to mild reductions in mitochondrial function (Fig. 2C). Similar to alanine, proline levels increased steadily over time in *Polg* mice, whereas levels were maintained in WT mice (Fig. 2D). Proline metabolism is involved in a redox coupling and regulatory system where proline and pyrroline-5-carboxylate serve as redox couples that can influence NAD^+^:NADH and NADP^+^:NADPH ratios (*19*–*21*). Proline can also be oxidized indirectly via glutamate and α-ketoglutarate in the TCA cycle, and these catabolic fluxes decrease progressively with complex I loss. Alanine and proline did not accumulate in WT mice even after 11 months, suggesting that these alterations are specific to progressive mitochondrial dysfunction rather than normal aging.

In contrast to the steady increase observed with alanine and proline, serine and histidine abundances remained consistently elevated in *Polg* plasma from 3 months onward (Fig. 2, E and F). On the other hand, glycine levels were largely unchanged between *Polg* and WT mice (Fig. 2G). Given the importance of NAD^+^ regeneration in de novo serine synthesis, this result is unexpected (*22*), and these changes likely reflect reduced catabolic flux through mitochondrial one-carbon metabolism (*6*, *23*). Methionine, another amino acid associated with one-carbon metabolism, was generally unchanged between WT and *Polg* mice (Fig. 2H).

Unexpectedly, branched-chain amino acids (BCAAs) such as leucine were unchanged between WT and *Polg* mice (Fig. 2I and fig. S1, A and B). BCAA catabolic flux generates significant NADH and is sensitive to hypoxic stress (*24*); however, reduced protein synthesis could balance this effect (*25*). Asparagine is another amino acid noted to increase during mitochondrial stress (*26*), and levels were generally higher in *Polg* mouse plasma (Fig. 2J). In contrast, the levels of aspartate, an amino acid whose levels are dependent on mitochondrial function (*27*, *28*), were mostly unchanged between *Polg* and WT mice (fig. S1C). Plasma concentrations of other amino acids were either unaltered or only significantly different at the last time point (fig. S1, D to J).

### Tissue-specific alterations in metabolites

**Fig. 3.**
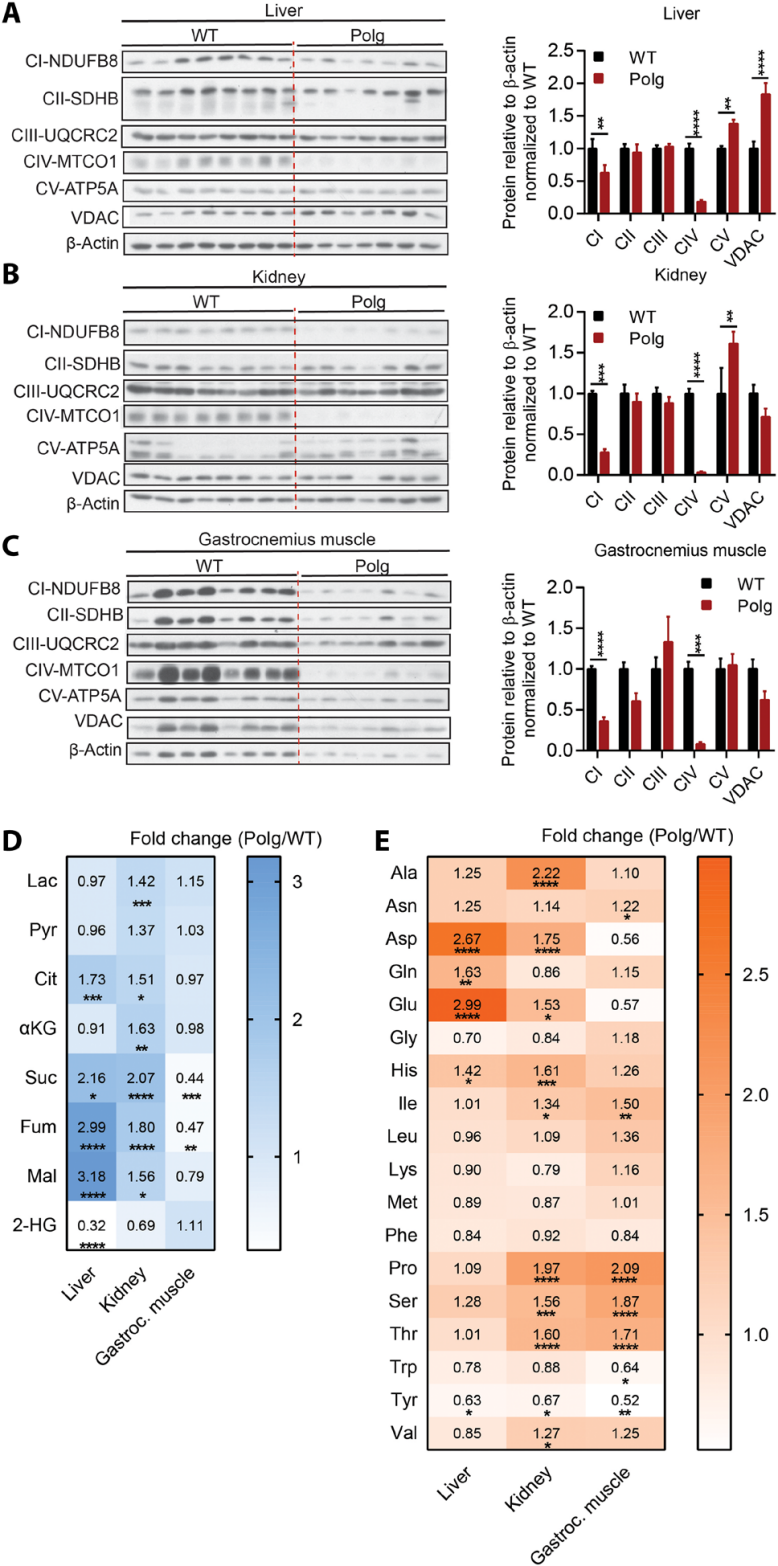
*Polg* mice exhibit tissue-specific alterations in metabolites. Western blot analysis of lysates and protein band quantification (relative density of protein to β-actin) from WT and *Polg* liver (**A**), kidney (**B**), and gastrocnemius muscle (**C**) targeting subunits of the ETC and voltage-dependent anion channel (VDAC) protein. (**D**) Heatmap representing average fold change of lactate, pyruvate, 2-HG, and TCA cycle intermediates [citrate (Cit), α-ketoglutarate, succinate (Suc), fumarate (Fum), and malate (Mal)] of *Polg* liver, kidney, and gastrocnemius muscle relative to WT tissue at 12 months of age. Refer to table S2 for tissue metabolite abundance. (**E**) Heatmap representing average fold change of amino acids in *Polg* liver, kidney, and gastrocnemius muscle relative to WT tissue at 12 months of age. Refer to table S3 for tissue amino acid concentration. Two-sided Student’s *t* test for each comparison with adjustment of multiple comparisons (Holm-Sidak method) (A to C). Two-way ANOVA with no adjustment for multiple comparisons (D and E). Data are means ± SEM of *n* = 7 to 8 animals per group. *P < 0.05, **P < 0.01, ***P < 0.001, and ****P < 0.0001.

The marked changes in circulating organic and amino acids led us to measure protein expression of the ETC complexes in the liver, kidney, and skeletal muscle (gastrocnemius) at the termination of the study, as these tissues are major contributors to the interorgan metabolite pool and exchange. We observed a significant reduction in key subunits of complex I and IV protein in the liver, kidney, and skeletal muscle (gastrocnemius) (Fig. 3, A to C). In contrast to the decrease in complexes I and IV, we detected no significant alteration in complex II and III subunits within these tissues (Fig. 3, A to C), while complex V was either increased or unchanged (Fig. 3, A to C). Overall, these data corroborate previously published data indicating that the primary impact of dysfunctional *Polg* encompasses a specific reduction in complexes I and IV (*29*–*31*), which are predominantly encoded from the mitochondrial genome. The selective loss of the ETC complexes is consistent with the larger increase in plasma TCA intermediates such as α-ketoglutarate and malate whose catabolism is directly NAD^+^ dependent (Fig. 1, H and K). By contrast, there was a more modest difference in the complex II–dependent intermediates, succinate and fumarate (Fig. 1, I and J).

We also quantified metabolite abundances across these tissues. Notably, lactate and pyruvate were significantly increased in the kidney only (Fig. 3D). In addition, there was a significant increase in succinate, fumarate, and malate in *Polg* liver and kidney, whereas succinate and fumarate were reduced in skeletal muscle (Fig. 3D). 2-HG levels were significantly decreased in *Polg* liver compared to WT (Fig. 3D), potentially due to altered NAD^+^/NADH or lactate dehydrogenase activity. The discrepancy in local and circulating TCA cycle intermediates likely reflects the tissue-specific origin and handling (anabolic versus catabolic) of each metabolite. For example, succinate is metabolized by distinct tissues and thus may show different changes locally and systemically.

Amino acids were altered in distinct tissues as well. Aspartate, glutamate, and glutamine accumulated significantly in the liver (Fig. 3E). Kidney and skeletal muscle were elevated in isoleucine, proline, serine, and threonine but showed reduced tyrosine abundance (Fig. 3E). Notably, alanine and lactate were markedly elevated in plasma but showed no significant accumulation in liver or skeletal muscle, which is suggestive of increased Cori and Cahill cycling between these tissues in *Polg* mice (fig. S2A). Their accumulation in the kidney highlights the importance of mitochondrial function in the renal system with respect to bioenergetics, gluconeogenesis, and nitrogen handling.

### Increased glycolysis and gluconeogenesis

**Fig. 4.**
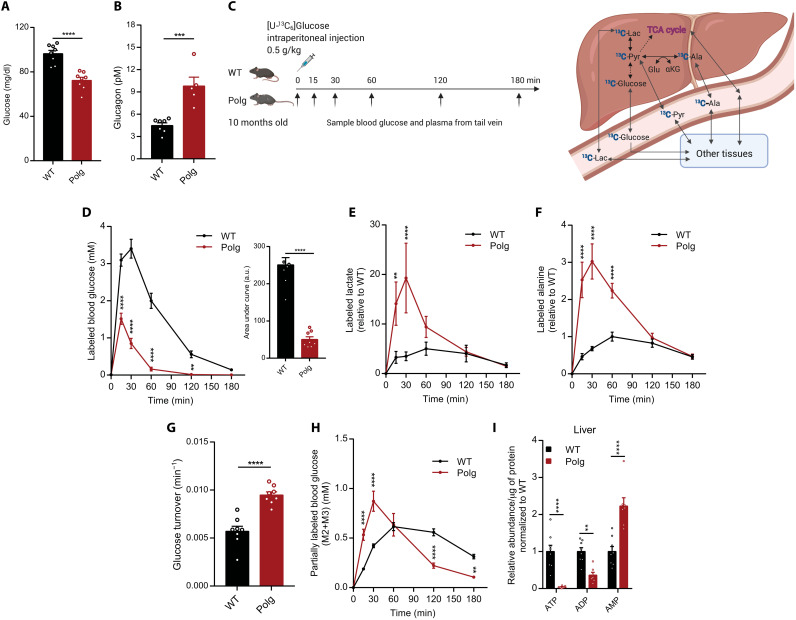
*Polg* mice exhibit hypoglycemia and increased Cori and Cahill cycling. Plasma glucose (**A**) and glucagon (**B**) levels in WT and *Polg* mice measured in fasted state. (**C**) Schematic of [U-^13^C_6_]glucose tracing experiment and how the ^13^C label is incorporated into newly synthesized pyruvate, alanine, lactate, and glucose. (**D**) Levels of ^13^C-labeled glucose and quantitation of area under the curve (a.u., arbitrary units). Labeled glucose was calculated by multiplying the fraction of glucose derivative M+5 with the plasma glucose concentration. Levels of ^13^C-labeled lactate (**E**) and alanine (**F**) (1-M0 fraction multiplied with metabolite abundance) relative to internal standard in plasma over time. Values are normalized to WT, and maximum WT value is set to 1. (**G**) Glucose turnover rate from intraperitoneal bolus injection. (**H**) Levels of partially labeled glucose (sum of M+2 and M+3 isotopomers of 370 *m*/*z* fragment) in plasma over time. (**I**) Abundance of nucleotide phosphates relative to internal standard in liver at 12 months of age. Abundances were normalized to μg protein per tissue. Refer to table S4 for tissue metabolite abundance. Two-sided Student’s *t* test for each comparison with no adjustment for multiple comparisons. Data are means ± SEM of *n* = 5 to 8 animals per group. *P < 0.05, **P < 0.01, ***P < 0.001, and ****P < 0.0001.

Given the profound changes in central carbon metabolism observed in *Polg* mice, we next sought to assess glucose flux and turnover in these animals. Similar to patients diagnosed with *POLG* mutations (*32*, *33*), *Polg* mice were hypoglycemic with fasting blood glucose levels that were significantly decreased relative to WT mice (Fig. 4A). In addition, in response to acute glucose challenge, plasma glucose cleared more rapidly in *Polg* compared to WT mice (fig. S3A). Consistent with hypoglycemia, fasting plasma glucagon levels were significantly increased in *Polg* mice compared to WT mice (Fig. 4B). On the other hand, insulin levels were not different between WT and *Polg* mice (fig. S3B).

To better quantify how glucose was metabolized differently in these mice, we administered uniformly labeled ^13^C [U-^13^C_6_]glucose to mice aged 10 months via intraperitoneal injection and collected plasma from the tail vein thereafter (Fig. 4C). Again, we observed that labeled glucose was more quickly metabolized by tissues, as reflected by a rapid decline and lower levels of circulating labeled glucose in *Polg* mice relative to WT (Fig. 4D). Concomitantly, we observed a significant increase in levels of labeled downstream metabolites such as lactate, pyruvate, and alanine in plasma, indicating a rapid uptake and metabolism of glucose in *Polg* mice (Fig. 4, E and F, and fig. S3C). The glucose turnover rate from the intraperitoneal bolus injection was significantly increased in *Polg* mice relative to WT (Fig. 4G). The labeling pattern of glucose can also reflect gluconeogenic flux (fig. S3D), and we observed increases in the relative abundance of partially labeled glucose indicative of elevated gluconeogenesis fragments (Fig. 4H and fig. S3, E to G). Consistent with the elevated glucagon (Fig. 4B), we also observed that *Polg* mice had higher expression of pyruvate carboxylase and phosphoenolpyruvate carboxykinase, key enzymes in the gluconeogenesis pathway (fig. S3H). To gain further insight into the energy state of these tissues, we measured levels of nucleotide phosphates in the liver and found that ATP levels were markedly reduced in *Polg* tissue, even in fed state (Fig. 4I). The significant reduction of ATP and increase in adenosine 5′-monophosphate (AMP) in *Polg* liver are consistent with increased gluconeogenesis to support the demand for Cori and Cahill cycling, further highlighting the importance of hepatic metabolism in regulating the whole-body energy status. Together, these data indicate that *Polg* mice exhibit an interesting glycolytic/gluconeogenic phenotype, likely to compensate for the lack of mitochondrial respiratory capacity.

### Altered nitrogen metabolism

**Fig. 5.**
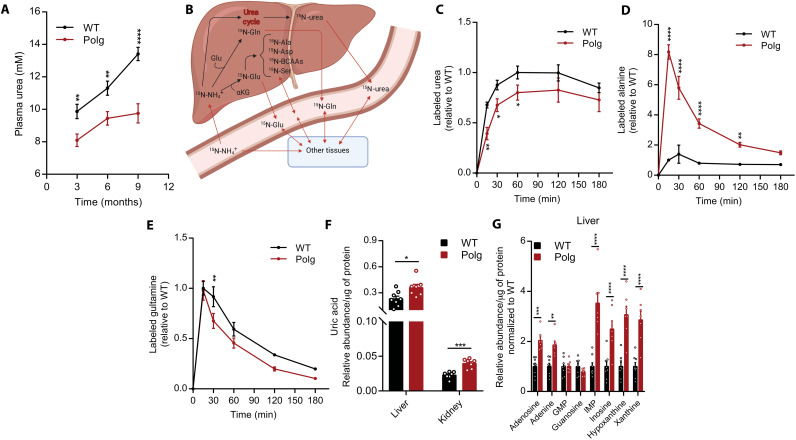
Altered nitrogen handling in *Polg* mice. (**A**) Concentration of plasma urea in WT and *Polg* mice at 3, 6, and 9 months of age. (**B**) Schematic of how ^15^N label from ammonium chloride (^15^N-NH_4_Cl) is incorporated into newly synthesized urea, glutamine, glutamate, and other metabolites. Levels of ^15^N-labeled urea (**C**), alanine (**D**), and glutamine (**E**) in plasma relative to internal standard over time. Values are normalized to WT, and maximum WT value is set to 1. (**F**) Abundance of uric acid relative to internal standard in liver and kidney at 12 months of age. Abundances were normalized to μg protein per tissue. (**G**) Abundance of purine catabolism intermediates relative to internal standard in liver at 12 months of age. Abundances were normalized to μg of protein per tissue. IMP, inosine 5′-monophosphate. GMP, guanosine 5’-monophosphate. Refer to table S5 for tissue metabolite abundance. Two-sided Student’s *t* test for each comparison with no adjustment for multiple comparisons. Data are means ± SEM of *n* = 6 to 8 animals per group. *P < 0.05, **P < 0.01, ***P < 0.001, and ****P < 0.0001.

Mitochondria and TCA intermediates are also associated with the urea cycle, and *Polg* mice exhibited significant alterations of several amino acids in plasma, most notably alanine and proline. Next, we assessed how nitrogen handling is altered in *Polg* mice. *POLG* mutations result in disorders that span a continuum of phenotypes, including liver dysfunction and failure (*34*, *35*). Notably, we observed that *Polg* mice had significantly reduced urea levels in plasma, suggesting impairment of the urea cycle (Fig. 5A). To explore how urea and amino acid nitrogen metabolism was altered in *Polg* mice, we administered ^15^N-ammonium chloride (NH_4_Cl) via intraperitoneal injection and collected plasma from the tail over 3 hours (Fig. 5B and fig. S4A). We observed a decrease in urea enrichment from ^15^N-NH_4_Cl, consistent with impaired urea cycling (Fig. 5C). Although there was no significant difference in ^15^N glutamate abundance (fig. S4B), we observed a substantial increase in labeled alanine in the plasma of *Polg* mice (Fig. 5D). We also detected elevated ^15^N enrichment of isoleucine (fig. S4C). The considerable labeling on alanine from ^15^N-NH_4_Cl indicates that flux through alanine transaminase is significantly elevated in *Polg* mice and matches our findings with [U-^13^C_6_]glucose (fig. S4D). Other metabolites either were not significantly different between WT and *Polg* mice (fig. S4, E and F) or had minimal enrichment from ^15^N-NH_4_Cl (fig. S4, G and H), highlighting the distinct impact of mitochondrial dysfunction on alanine metabolism. Glutamine is another important nitrogen pool in plasma, as glutamine synthetase incorporates nitrogen from ammonium into the glutamine pool. Incorporation of ^15^N-NH_4_Cl into plasma glutamine was slightly reduced in *Polg* mice (Fig. 5E). Overall, these results suggest that mitochondrial dysfunction in *Polg* mice impairs nitrogen incorporation into urea, which further contributes to alanine accumulation in *Polg* mice.

Another mechanism for excreting nitrogenous waste is via purine degradation to uric acid (fig. S4I). While uric acid was slightly elevated in plasma (fig. S4J), we detected a significant increase in uric acid within the liver and kidney of *Polg* mice (Fig. 5F). Furthermore, there was a significant increase in various purine metabolites in *Polg* liver (Fig. 5G) and, to a lesser extent, in kidney (fig. S4K). *Polg* livers also exhibited increased expression of AMP deaminase 3 and xanthine dehydrogenase (fig. S4L), along with higher AMP levels (Fig. 4I). Together, the increased purine catabolism combined with impaired urea cycle suggests that mitochondrial dysfunction in *Polg* mice also affects nitrogen metabolism.

### Dysregulated lipid metabolism

**Fig. 6.**
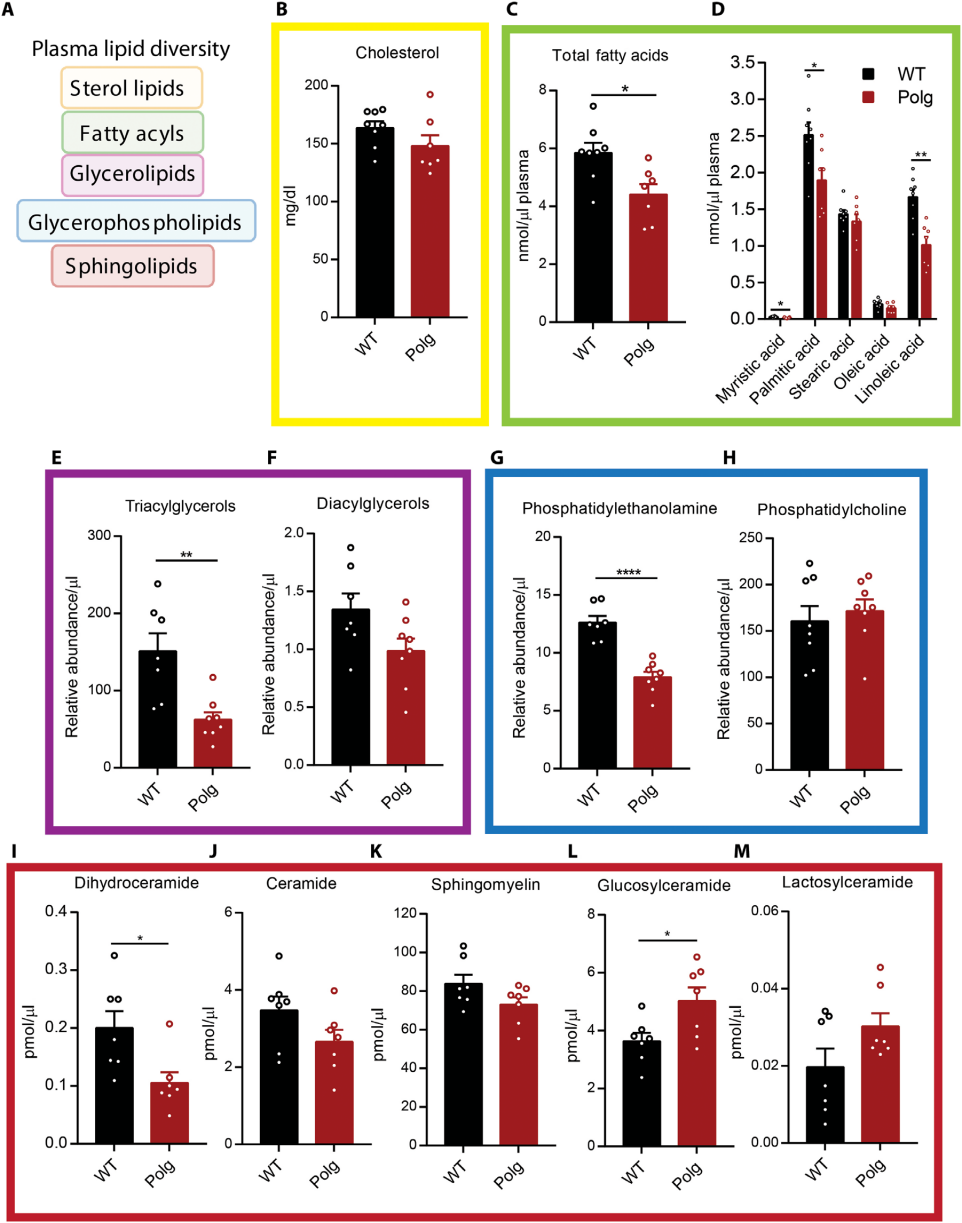
Dysregulated lipid metabolism in *Polg* mice. (**A**) Schematic of major plasma lipids. (**B**) Concentration of plasma cholesterol in WT and *Polg* mice. Concentration of total (**C**) and individual (**D**) plasma fatty acids in WT and *Polg* mice. Abundance of plasma triacylglycerols (**E**) and diacylglycerols (**F**), phosphatidylethanolamine (PE) (**G**), and phosphatidylcholine (PC) (**H**) relative to internal standard per microliter of plasma in WT and *Polg* mice. Concentration of plasma dihydroceramide (**I**), ceramide (**J**), sphingomyelin (**K**), glucosylceramide (**L**), and lactosylceramide (**M**) in WT and *Polg* mice. Two-sided Student’s *t* test for each comparison with no adjustment for multiple comparisons. Data are means ± SEM of *n* = 7 to 8 animals per group. *P < 0.05, **P < 0.01, and ****P < 0.0001.

*Polg* mice have reduced body fat compared to WT mice and gain minimal weight even when fed a high-fat diet (*36*, *37*). Beyond these changes in adiposity, the lipid composition of *Polg* mice has not been explored in detail. To this end, we quantified various lipid species in our *Polg* and WT cohorts (Fig. 6A). The levels of cholesterol, the most abundant lipid in plasma, were unaltered in *Polg* plasma (Fig. 6B). In contrast, there was a significant reduction in total fatty acids (Fig. 6C). The reduction in fatty acids was not uniform across the different species, with significant decreases only in myristic acid, palmitic acid, and the essential fatty acid linoleic acid (Fig. 6D). These changes reflect potential impacts on fatty acid synthesis as well as absorption from the gut from severe mitochondrial dysfunction. We also observed a significant decrease in triacylglycerols but not diacylglycerols in *Polg* plasma (Fig. 6, E and F, and fig. S5, A and B). Analysis of total circulating glycerophospholipids revealed a decrease in phosphatidylethanolamine (PE) (Fig. 6G and fig. S5C), but no change in phosphatidylcholine (PC) in *Polg* mice (Fig. 6H and fig. S5D). Notably, PE synthesis occurs in the mitochondria (*38*) in addition to the endoplasmic reticulum (ER) (*39*). On the other hand, PC synthesis is dependent on the ER and Golgi membranes in most tissues, although the liver and adipocytes are capable of methylating PE to PC in ER-mitochondria membrane domains (*40*).

Sphingolipids are an important class of bioactive lipids that incorporate metabolic signals from fatty acids/acyl-coenzyme As (CoAs) and amino acids. We observed no differences in the sphingoid bases, sphinganine and sphingosine, between *Polg* and WT mice (fig. S5, E and F). In contrast, there was a significant reduction in plasma dihydroceramides in *Polg* plasma (Fig. 6I and fig. S5G), while ceramides remained unchanged (Fig. 6J and fig. S5H). This decrease in dihydroceramides is suggestive of reduced de novo sphingolipid biosynthesis, as dihydroceramides are the direct product of serine palmitoyltransferase (SPT). On the other hand, ceramides are more readily salvaged from sphingomyelin, which was also similarly abundant in *Polg* and WT mouse plasma (Fig. 6K). These data are consistent with plasma cholesterol results, as cholesterol, sphingomyelin, and ceramides are abundant components of lipoproteins. Last, we observed a slight increase in plasma hexosylceramides but more varied results with less abundant lactosylceramides (Fig. 6, L and M, and fig. S5J). In various sphingolipid classes, we observed a specific reduction in species containing acyl chains of C20:0 or longer in *Polg* mice but the reverse for shorter chain length species (fig. S5, H to J). These data suggest that fatty acid elongation or the activity of specific ceramide synthases is compromised in *Polg* mice.

**Fig. 7.**
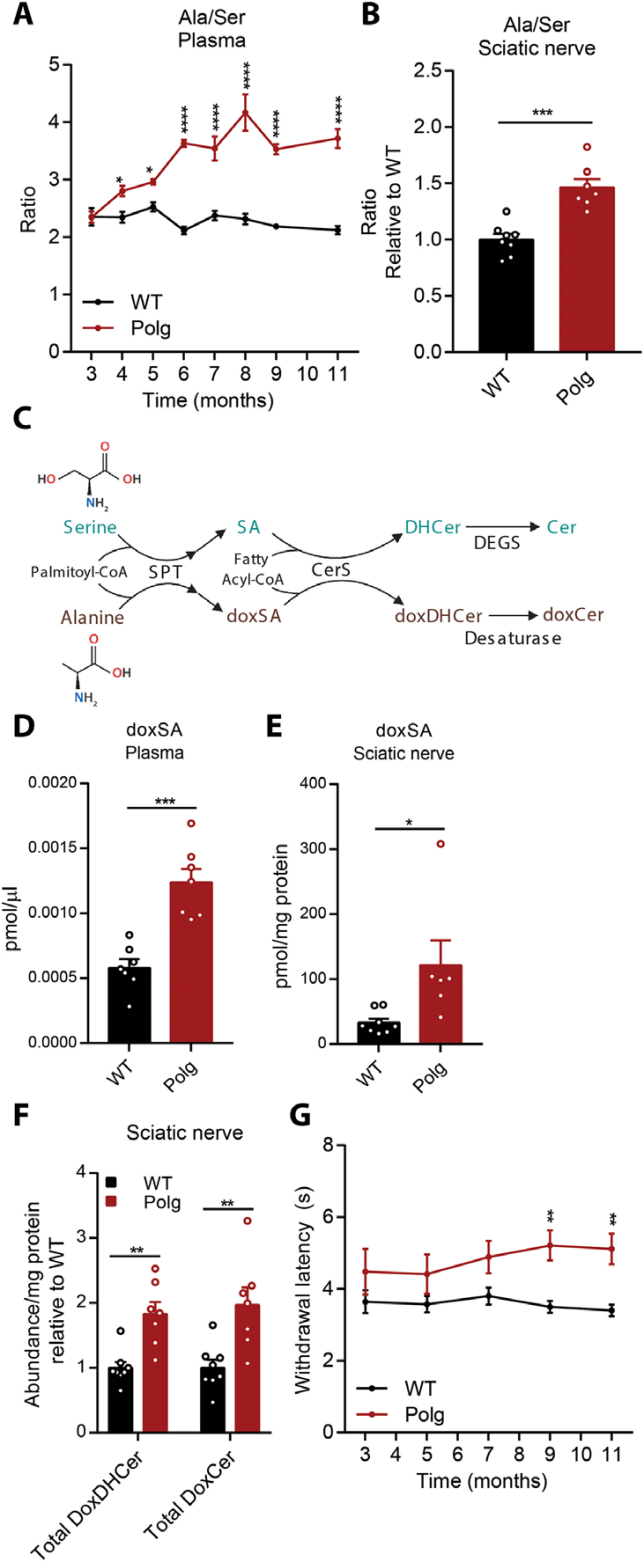
*Polg* mice accumulate doxSLs and develop thermal hypoalgesia. Ratio of serine:alanine (Ser/Ala) in plasma from 3 to 11 months of age (**A**) and sciatic nerve (**B**). (**C**) Schematic describing synthesis of canonical sphingolipids from serine (teal) and doxSLs from alanine (brown) via promiscuous SPT activity. Ceramide synthase (CerS) produces 1-dihydroceramides (DHCer) or 1-deoxydihydroceramides (doxDHCer). Dihydroceramide desaturase (DEGS) forms ceramides (Cer), and desaturases such as fatty acid desaturase 3 (FADS3) form 1-deoxyceramides (doxCer) (*74*). Concentration of 1-deoxysphinganine (doxSA) in plasma (**D**) and sciatic nerve (**E**) in WT and *Polg* mice. (**F**) Abundance of sciatic nerve doxDHCer and doxCer relative to internal standard per milligram of protein per tissue in WT and *Polg* mice. (**G**) Paw heat withdrawal response latency measured every 2 months in WT and *Polg* mice. Two-way ANOVA (A and G) or two-sided Student’s *t* test (B and D to F) for each comparison with no adjustment for multiple comparisons. Data are means ± SEM of *n* = 7 to 8 animals per group. *P < 0.05, **P < 0.01, ***P < 0.001, and ****P < 0.0001.

The sustained increase in plasma alanine in *Polg* mice led to a progressive increase in the alanine:serine ratio (Fig. 7A), which was also elevated in the sciatic nerve (Fig. 7B). Alterations in the balance of serine and alanine drive the accumulation of doxSLs due to promiscuous activity of SPT (Fig. 7C) (*41*, *42*). DoxSLs accumulate in various contexts, including HSAN1 (*14*, *43*), metabolic syndrome (*15*), macular telangiectasia (MacTel) (*44*), dietary serine restriction (*45*), and aging (*46*). 1-Deoxysphinganine (doxSA) was significantly elevated in the plasma and sciatic nerve of *Polg* mice (Fig. 7, D and E), and both 1-deoxy dihydroceramide (doxDHCer) and 1-deoxyceramide (doxCer) pools were significantly increased in *Polg* sciatic nerve (Fig. 7F and fig. S6, A and B). Assessment of the canonical sphingolipids including ceramide, sphingomyelin, glucosylceramides, and lactosylceramides revealed no significant differences between WT and *Polg* mice (fig. S6, C to H).

### Development of thermal hypoalgesia

The elevation in alanine and doxSLs in *Polg* is intriguing because these lipids have been shown to cause HSAN1 (*14*), an axonal neuropathy marked by thermal hypoalgesia and driven by gain-of-function SPT variants that preferentially use alanine as a substrate. Furthermore, doxSLs have been associated with diabetic peripheral neuropathy (*15*, *16*) and chemotherapy-induced peripheral neuropathy (*17*). Peripheral neuropathy is common in mitochondrial diseases, although the clinical manifestation spans a wide spectrum (*47*). We specifically hypothesized that the increase in circulating alanine drives doxSL synthesis and transport to drive thermal hypoalgesia in *Polg* mice. Feeding a serine- and glycine-free diet to C57BL/6 mice drives both doxSL accumulation and thermal hypoalgesia measured by hot plate assay (*44*). *Polg* mice presented with a slight increase in thermal latency compared to WT mice through 7 months of age, and thermal hypoalgesia became significant at 9 months as determined by Hargreaves test (Fig. 7G). On the other hand, we observed no change in nerve conduction velocity (NCV) or mechanical nociception in *Polg* mice (fig. S6, I and J). This finding is significant given the specificity of the neuropathic phenotype, as doxSLs are genetically linked to HSAN1/thermal hypoalgesia, while other sphingolipids (e.g., S1P) have been implicated in distinct neuropathic phenotypes (*48*). Furthermore, by linking mitochondrial dysfunction to alanine and doxSL accumulation, we highlight a distinct mechanism through which mitochondrial defects biochemically drive the accumulation of toxic lipids that are associated with thermal hypoalgesia (Fig. 7G).

## DISCUSSION

Here, we comprehensively characterized the metabolic compensation that occurs during progressive loss of mitochondrial function in the *Polg* mouse model. We longitudinally profiled metabolites from both *Polg* and WT mice and observed an accumulation of alanine, pyruvate, and lactate as well as several TCA intermediates and other amino acids. The accumulation of these metabolites is a hallmark of mitochondrial respiratory chain disorders and is consistent with defects associated with loss of complexes I and IV, which are predominantly encoded by the mitochondrial genome. The time course data presented here not only agree with published clinical data (*18*, *49*–*51*) but also reveal the order and prioritization of the metabolic alterations that occur in response to cumulative mitochondrial stress. The increase in alanine is particularly striking considering how its accumulation preceded lactate and pyruvate, and the lack of significant changes in normal aging mice highlights the specificity of these changes to accumulated mitochondrial defects.

The hypoglycemia and increased glycolytic flux in *Polg* mice complement published data indicating that *Polg* mice rely on glycolysis for energy production (*52*). Administration of [U-^13^C_6_]glucose allowed us to demonstrate high rates of Cori and Cahill cycling via lactate and alanine, respectively, which also correlated with high fasting glucagon levels compared to WT mice. The reduction in circulating glucose and increased circulating lactate and alanine further support the notion of increased gluconeogenic flux in *Polg* mice, which has also been reported in patients with mitochondrial disease (*53*, *54*). Furthermore, patients with *POLG* mutations have been reported to be hypoglycemic and glucagon insensitive (*32*, *33*). The role of endocrine hormones in regulating glucose homeostasis in the context of *POLG*-driven mitochondrial impairment and stress will be insightful to identify therapeutic targets for mitochondrial disease patients. In contrast to that observed in hematopoietic stem cells and T cells with defective mitochondria (*55*, *56*), we observed reduced levels of 2-HG in liver of *Polg* mice (Fig. 3D). As 2-HG predominantly arises from lactate dehydrogenase activity (*57*), these data suggest that hepatic 2-HG production is reduced due to altered NAD^+^/NADH state and gluconeogenic flux of hepatic lactate dehydrogenase in *Polg* mice.

While alanine is a known gluconeogenic substrate (*58*), the role of dysfunctional nitrogen metabolism in altering amino acid pools warrants consideration. The impairment of mitochondrial metabolism will affect the urea cycle, as evidenced by our ^15^N-NH_4_Cl tracing, which not only primarily tracks amino acid and transaminase metabolism but also efficiently labels blood urea. Impairments in the TCA and urea cycle may compromise nitrogen handling, driving the animal to increase flux through alternate pathways such as pyruvate transamination to alanine or purine degradation. Notably, a recent mitochondrial genome sequencing study identified several alleles associated with pathogenic gout (*59*), highlighting the clinical relevance of this pathway alteration. The increase in AMP in *Polg* mice is intriguing as it suggests potential activation of AMP-activated protein kinase (AMPK), which may be engaged as a mechanism to reverse the bioenergetic stress caused from the cumulative effects of the *POLG* mutation. As AMPK regulates a diverse set of metabolic pathways to restore ATP levels (*60*), it would be interesting to understand the role of AMPK in a model of mitochondrial dysfunction such as the *Polg* mice.

Mitochondrial dysfunction in respiratory chain diseases has been shown to alter a wide range of lipid species that are suggestive of disrupted fatty acid oxidation and/or impaired biosynthesis (*49*, *51*, *61*). Our survey of the plasma lipidome in *Polg* mice revealed a reduction in multiple classes of lipids including fatty acids, glycerolipids, glycerophospholipids, and sphingolipids. While the reduction of some (e.g., glycerolipids and glycerophospholipid) is not consistent with previously published data from humans diagnosed with primary mitochondrial disease (*49*, *51*, *61*), the discrepancy may point toward a different mechanism for the lipid alteration. Plasma triglyceride content is heavily dependent on diet. In the case of *Polg* mice, the *Polg* D257A mutation has been shown to limit fat absorption by the intestine, leading to attenuated weight gain even on a high-fat diet (*37*). The observed reduction in some lipids could be due to reduced intestinal absorption, which, in turn, could drive shifts in the acyl chain distributions of ceramides, sphingomyelin, and glucosylceramides.

We also observed the accumulation of doxSLs in *Polg* mice, which ultimately develop thermal hypoalgesia. While previous work has focused on the role of SPT variants or serine availability in doxSL accumulation (*43*–*45*), we identify a mitochondria-driven mechanism whereby elevated alanine drives increased levels of doxSLs. Elevated doxDHCer and 1-deoxysphingosine (doxSO) have been detected in a small subset of individuals with primary mitochondrial disease and peripheral neuropathy (*62*). Others have shown that doxSLs increase in the central nervous system with age (*63*). Mechanistically, doxSLs have been shown to disrupt neuronal cytoskeleton structures (*14*), reduce neurite length (*14*), cause mitochondrial fragmentation (*64*), and induce the collapse of the ER membrane (*65*). Here, we demonstrate that mitochondrial defects can directly drive changes in membrane lipid metabolism. While bioenergetic defects also likely contribute to this peripheral neuropathy phenotype, doxSL accumulation in the context of mitochondrial dysfunction may perturb multiple membrane-associated cellular functions that are deleterious for nervous system function.

Last, our results highlight the utility of *Polg* mice in deciphering how to design diets that mitigate phenotypes associated with mitochondrial stress. By performing longitudinal metabolomic and phenotypic measurements during aging of *Polg* mice, we identified key pathway alterations that lead to neuropathy. The use of stable isotope tracers in such models allowed us to decipher key pathway alterations driving these phenotypes. Because these findings are highly dependent on amino acid intake, they can be leveraged to understand how dietary (or pharmacological) interventions could mitigate the deleterious effects of both mild (early) and severe (late) mitochondrial dysfunction.

## MATERIALS AND METHODS

### *Polg*^D257A^ mtDNA mutator mouse

Mouse handling and care followed the National of Institutes of Health’s *Guide for the Care and Use of Laboratory Animals*. The experimental protocols were approved by the University of California San Diego Institutional Animal Care and Use Committee. Twelve-week-old male *Polg*^D257A^ and WT mice were purchased from the Jackson Laboratory. Food and water were provided ad libitum.

### Plasma and tissue sampling

Blood samples were collected in fed state (unless otherwise noted) via tail bleed in EDTA-coated tubes (Sarstedt Inc.) and spun at 2000*g* for 5 min. The supernatant was transferred to a new Eppendorf tube and stored at −80°C until analysis. Tissues were collected in fed state in the morning (6 a.m.) when the mice were 12 months old. Tissues such as the liver, kidney, skeletal muscle, and sciatic nerve were freeze-clamped immediately upon collection using Wollenberger clamps, precooled to the temperature of liquid nitrogen, and stored at −80°C until further analysis.

### Polar metabolite analysis (gas chromatography–mass spectrometry)

Metabolites from 3 μl of plasma were extracted with 250 μl of −20°C methanol, with inclusion of 3 μl of 100 μM labeled (^13^C,^15^N) amino acid standard mix (Cambridge Isotope Laboratories Inc., MSK-CAA-1 or MSK-A2-1.2). The tubes were vortexed for 10 min and centrifuged at 16,000*g* at 4°C for 10 min. The supernatant was collected and dried under vacuum. Derivatization for polar metabolites was performed using a Gerstel MPS with 15 μl of 2% (w/v) methoxyamine hydrochloride (Thermo Fisher Scientific) in pyridine (incubated for 60 min at 45°C) and followed by 15 μl of *N*-tertbutyldimethylsilyl-*N*-methyltrifluoroacetamide (MTBSTFA) with 1% tert-butyldimethylchlorosilane (Regis Technologies) (incubated for 30 min at 45°C). Polar derivatives were analyzed by gas chromatography–mass spectrometry (GC-MS) using a DB-35MSUI column [30 m by 0.25 mm inner diameter (i.d.) by 0.25 m] installed in an Agilent 7890B GC interfaced with an Agilent 5977A MS. For metabolite separation, the GC oven was held at 100°C for 2 min followed by increasing the temperature to 320°C at a ramp rate of 10°C/min and held for 4 min. The eluates from the GC were subjected to electron impact ionization. MS scanning was performed over mass/charge ratio (*m*/*z*) range of 100 to 650. The MS source and quadrupole were held at 230° and 150°C, respectively.

Details on specific fragments are provided elsewhere (*66*). The % isotopologue distribution of each fatty acid and polar metabolite was determined by integration using an in-house MATLAB-based algorithm and corrected for natural abundance using the method described elsewhere (*67*).

### Glucose measurement and analysis

Plasma glucose levels were measured with a handheld glucometer device (Contour Next One, Ascensia Diabetes Care). For labeling measurements using GC-MS, glucose was derivatized by dissolving 3 μl of plasma in 50 μl of 2% (w/v) hydroxylamine hydrochloride in pyridine and incubating at 90°C for 60 min. After the incubation, 100 μl of propionic anhydride was added and samples were incubated at 60°C for 30 min. Last, samples were evaporated at 60°C and dissolved in 100 μl of ethyl acetate.

Glucose derivatives were analyzed by GC-MS as described above with the following method: the GC oven was held at 100°C for 1 min followed by increasing the temperature to 280°C at a ramp rate of 20°C/min and held for 4 min. Details on specific fragments are provided elsewhere (*68*).

### Polar metabolite analysis (liquid chromatography–MS)

Details below were adapted from MacKay *et al.* (*69*) with some modifications. Metabolites from 15 μl of plasma were extracted with 400 μl of −20°C 5:3:2 acetonitrile:methanol:water solution, with inclusion of 10 μl of norvaline (100 μg/ml). The tubes were vortexed for 5 min and centrifuged at 16,000*g* at 4°C for 5 min. The supernatant was then transferred for analysis.

For metabolite extraction from tissue, 10 to 20 mg of frozen tissue were homogenized with the Precellys Evolution Homogenizer (Bertin Technologies Inc.) at 6800 rpm in three cycles of 20 s, with 30-s pause in between each cycle, in 1 ml of −20°C 5:3:2 acetonitrile:methanol:water solution, with inclusion of 10 μl of labeled (^13^C) NAD^+^ (100 μg/ml; Cambridge Isotope Laboratories Inc., CLM-10671-PK) and 10 μl of norvaline (100 μg/ml; Sigma-Aldrich, N7502). Homogenate (20 μl) was dried under air and redissolved in 20 μl of M-PER buffer (Thermo Fisher Scientific, 78501) for protein estimation with bicinchoninic acid (BCA) assay. The tubes were centrifuged at 16,000*g* at 4°C for 5 min. The supernatant was then transferred for analysis.

Q Exactive Orbitrap MS with a Vanquish Flex Binary UHPLC system (Thermo Fisher Scientific) was used with an iHILIC-(P) Classic, 150 mm by 2.1 mm, 5-μm particle, 200-Å (Hilicon) column at 45°C. Sample (2 to 5 μl) was injected. Chromatography was performed using a gradient of 20 mM ammonium carbonate, adjusted to pH 9.4 with 0.1% ammonium hydroxide (25%) solution (mobile phase A) and 100% acetonitrile (mobile phase B), both at a flow rate of 0.2 ml/min. The liquid chromatography (LC) gradient ran linearly from 80 to 20% B from 2 to 17 min and then from 20 to 80% B from 17 to 18 min and then held at 80% B from 18 to 25 min.

Plasma and liver samples were analyzed in negative mode using spray voltage of 3.25 kV. Kidney samples were analyzed in polarity switching mode, with the positive mode using spray voltage of 4.25 kV. For both positive and negative mode, auxiliary gas flow was 15 arbitrary units and sheath gas flow was 25 arbitrary units, with a capillary temperature of 275°C. Full MS (scan range of 75 to 1000 *m*/*z*) was used at 70,000 resolution with 1 × 10^6^ automatic gain control and a maximum injection time of 100 ms. Metabolite retention times were verified using standards, and in addition, a subset of samples was analyzed in MS2 (top 6) mode to increase confidence in metabolite identification. Mass accuracy was below 5 parts per million (ppm) for all analytes. Data were analyzed using EI-Maven software, and peaks were normalized to norvaline or labeled (^13^C) NAD^+^ internal standard. Refer to table S6 for ion *m*/*z* and retention time.

### Fatty acid and cholesterol analysis

Fatty acid and cholesterol were extracted from 10 μl of plasma with 250 μl of methanol, 250 μl of saline, and 500 μl of chloroform, with inclusion of [^2^H_31_]palmitate (Cambridge Isotope Laboratories Inc., DLM-215-1) and [^2^H_7_]cholesterol (Cambridge Isotope Laboratories Inc., DLM-3057-PK) as internal standards. The tubes were vortexed for 10 min and centrifuged at 16,000*g* at 4°C for 10 min to allow phase separation. The lower organic layer was collected and dried under air at room temperature. The organic phase was derivatized to form fatty acid methyl esters (FAMEs) via addition of 500 μl of 2% H_2_SO_4_ in methanol and incubation at 50°C for 2 hours. FAMEs were extracted via addition of 100 μl of saturated salt solution and 500 μl of hexane. FAMEs were then analyzed using a Select FAME column (100 m by 0.25 mm i.d.) installed in an Agilent 7890A GC interfaced with an Agilent 5975C MS. GC oven was held at 80°C for 1 min after injection, increased to 170°C by 20°C/min, increased to 188°C by 1°C/min, and finally increased to 250°C by 20°C/min and held for 10 min.

After FAME analysis, the samples were dried under air and converted to cholesterol trimethylsilyl (TMS) derivatives by adding 30 μl of *N*-methyl-*N*-( trimethylsilyl) trifluoroacetamide (Regis Technologies) and incubating at 37°C for 30 min. Cholesterol derivatives were analyzed by GC-MS using a DB-35MS column (30 m by 0.25 mm i.d. by 0.25 m) installed in an Agilent 7890B GC interfaced with an Agilent 5977B MS. The GC oven temperature was held at 150°C for 1 min, then to 260°C at 20°C/min, then held for 3 min, and further increased to 280°C at 10°C/min and held for 15 min, and finally ramped up to 325°C for a total run time of approximately 30 min. The MS source and quadrupole were held at 230° and 150°C, respectively, and the detector was operated in scanning mode, recording ion abundance in the range of 100 to 650 *m*/*z*.

### Lipid analysis

Lipids (glycerolipids and glycerophospholipids) were extracted from 10 μl of plasma with 750 μl of ice-cold 1:1 methanol/water solution and 500 μl of ice-cold chloroform with inclusion of EquiSPLASH (Avanti, Croda International Plc, 330731), C15 Glucosyl(β) Ceramide-d7 (d18:1-d7/15:0) (Avanti, Croda International Plc, 330729), and C15 Lactosyl(β) Ceramide-d7 (d18:1-d7/15:0) (Avanti, Croda International Plc, 330727) as internal standards. The tubes were vortexed for 5 min and centrifuged at 16,000*g* at 4°C for 5 min. The lower organic phase was collected, and 2 μl of formic acid was added to the remaining polar phase, which was reextracted with 1 ml of chloroform. Combined organic phases were dried, and the pellet was resuspended in 50 μl of isopropyl alcohol.

Q Exactive Orbitrap MS with a Vanquish Flex Binary UHPLC system (Thermo Fisher Scientific) was used with an Accucore C30, 150 mm by 2.1 mm, 2.6-μm particle (Thermo Fisher Scientific) column at 40°C. Sample (5 μl) was injected. Chromatography was performed using a gradient of 40:60 (v/v) water:acetonitrile with 10 mM ammonium formate and 0.1% formic acid (mobile phase A) and 10:90 (v/v) acetonitrile:2-propanol with 10 mM ammonium formate and 0.1% formic acid (mobile phase B), both at a flow rate of 0.2 ml/min. The LC gradient ran from 30 to 43% B from 3 to 8 min, then from 43 to 50% B from 8 to 9 min, then from 50 to 90% B from 9 to 18 min, and then from 90 to 99% B from 18 to 26 min, then held at 99% B from 26 to 30 min, before returning to 30% B in 6 min, and held for a further 4 min.

Lipids were analyzed in positive mode using spray voltage of 3.2 kV. Sweep gas flow was 1 arbitrary unit, auxiliary gas flow was 2 arbitrary units, and sheath gas flow was 40 arbitrary units, with a capillary temperature of 325°C. Full MS (scan range, 200 to 2000 *m*/*z*) was used at 70,000 resolution with 1 × 10^6^ automatic gain control and a maximum injection time of 100 ms. Data-dependent MS2 (top 6) mode at 17,500 resolution with automatic gain control set at 1 × 10^5^ with a maximum injection time of 50 ms was used. Data were analyzed using EI-Maven software, and peaks were normalized to Avanti EquiSPLASH internal standard. Refer to table S7 for ion transitions of lipid species.

For targeted sphingolipid analysis, 50 μl of plasma was extracted with 500 μl of −20°C methanol, 400 μl of saline, and 100 μl of water spiked with deuterated internal standards [100 pmol of D7-sphingosine (Avanti, Croda International Plc, 860657), 20 pmol of D7-sphinganine (Avanti, Croda International Plc, 860658), 2 pmol of D3-deoxysphinganine (Avanti, Croda International Plc, 860474), 200 pmol of C15 ceramide-d7 (d18:1-d7/15:0) (Avanti, Croda International Plc, 860681), 100 pmol of C13-dihydroceramide-d7 (d18:0-d7/13:0) (Avanti, Croda International Plc, 330726), 10 pmol of glucosylsphingosine (d18:1-d7) (Avanti, Croda International Plc, 860695), 100 pmol of glucosylceramide (d18:1-d7/15:0) (Avanti, Croda International Plc, 330729), 100 pmol of lactosylceramide (d18:1-d7/15:0) (Avanti, Croda International Plc, 330727), and 200 pmol of sphingomyelin (d18:1/18:1-d9) (Avanti, Croda International Plc, 791649)]. Chloroform (1 ml) was then added to the tubes. The tubes were vortexed for 5 min and centrifuged at 16,000*g* at 4°C for 5 min. The lower organic phase was collected, and 2 μl of formic acid was added to the remaining polar phase, which was reextracted with 1 ml of chloroform. Combined organic phases were dried and resuspended in 100 μl of 0.2% formic acid and 1 mM ammonium formate in methanol. Next, the tubes were sonicated in a bath sonicator for 10 min and spun at 16,000*g* for 10 min at 4°C.

For sphingolipid extraction from sciatic nerve, frozen tissue was homogenized with a ball mill (Retsch Mixer Mill MM 400) at 30 Hz for 3 min in 500 μl of −20°C methanol, 400 μl of ice-cold saline, and 100 μl of ice-cold MilliQ water, spiked with deuterated internal standards as described earlier. The mixture was then transferred into a 2-ml Eppendorf tube containing 1 ml of chloroform. The tubes were vortexed for 5 min and centrifuged at 16,000*g* at 4°C for 5 min. The lower organic phase was collected, and 2 μl of formic acid was added to the remaining polar phase, which was reextracted with 1 ml of chloroform. Combined organic phases were dried and resuspended in 50 μl of 0.2% formic acid and 1 mM ammonium formate in methanol. Last, the tubes were sonicated in a bath sonicator for 10 min and spun at 16,000*g* for 10 min at 4°C.

Ceramide species in the supernatant were then quantified via LC-MS [Agilent 6460 QQQ, MassHunter LC/MS Acquisition (v.B.08.02)]. Ceramides were separated on a C8 column (Spectra 3 μm C8SR 150 mm by 3 mm i.d., Peeke Scientific) as previously described (*70*). Mobile phase A was composed of 100% high-performance LC–grade water containing 2 mM ammonium formate and 0.2% formic acid, and mobile phase B consisted of 100% methanol containing 0.2% formic acid and 1 mM ammonium formate. The gradient elution program consisted of the following profile: 0 min, 82% B; 3 min, 82% B; 4 min, 90% B; 18 min, 99% B; 25 min, 99%; 27 min, 82% B; and 30 min, 82% B. Column reequilibration followed each sample and lasted 10 min. The capillary voltage was set to 3.5 kV, the drying gas temperature was 350°C, the drying gas flow rate was 10 liters/min, and the nebulizer pressure was 60 psi. Ceramide species were analyzed by selective reaction monitoring of the transition from precursor to product ions at associated optimized collision energies, and fragmentor voltages are provided elsewhere (*45*). Ceramide and dihydroceramide species were then quantified from spiked internal standards.

### Stable isotope tracing

For stable isotope tracing experiments, food was removed from cages at 5:00 p.m. the night before the study. Blood was collected via tail bleed at baseline and 15, 30, 60, 120, and 180 min after intraperitoneal injection of stable isotope tracer. Mice remained in their original cage with original bedding for the duration of the experiment.

Uniformly labeled ^13^C-glucose ([U-^13^C_6_]glucose) (Cambridge Isotope Laboratories Inc., CLM-1396-PK) was prepared as a solution (50 g/liter) in sterile phosphate-buffered saline (PBS). On the day of experiment, mice were weighed and the appropriate volume of [U-^13^C_6_]glucose solution was administered via intraperitoneal injection at 0.5 g/kg. Fifteen N-labeled ammonium chloride (^15^N-NH_4_Cl) (Cambridge Isotope Laboratories Inc., NLM-467-PK) was prepared as a solution (16.7 g/liter) in sterile PBS. On the day of experiment, mice were weighed and the appropriate volume of ^15^N-NH_4_Cl was administered via intraperitoneal injection at 0.167 g/kg.

Using the single-pool model, glucose turnover rate was calculated as described elsewhere (*71*): $\text{Metabolite turnover},k=\frac{\text{ln}\;E\;[t(1)]-\text{ln}\;E[t(2)]}{t_{2}-t_{1}}$, with *E* being the plasma enrichment from labeled glucose at *t*_1_ = 15 min and *t*_2_ = 180 min.

### Insulin and glucagon measurement

Insulin and glucagon levels were determined in fasted plasma using commercially available enzyme-linked immunosorbent assay (ELISA) kits from Mercodia Inc. [Insulin ELISA (10-1247-01) and Glucagon ELISA (10-1281-01)]. Concentrations were measured according to the manufacturer’s instructions.

### Protein extraction and immunoblotting

Tissue lysates (liver, whole kidney, and skeletal muscle) were generated from 10 to 20 mg of pieces of frozen tissue homogenized in cell lysis buffer [Cell Signaling Technology #9803; 20 mM tris-HCl (pH 7.5), 150 mM NaCl, 1 mM EDTA, 1 mM EGTA, 50 mM sodium fluoride, 1% Triton X-100, 2.5 mM sodium pyrophosphate, 1 mM β-glycerophosphate, 1 mM Na_3_VO_4_, and 10 nM calyculin A] supplemented with protease inhibitors (Roche, 11836170001). Tissues were mechanically homogenized for 30 s and left on ice for 10 min. Lysates were then centrifuged at 16,000*g* for 15 min at 4°C, and supernatant was transferred to a second microtube. Protein concentration was determined using a Pierce BCA protein assay kit (Thermo Fisher Scientific, 23225). SDS–polyacrylamide gel electrophoresis (PAGE) running samples were prepared and incubated either at room temperature, for resolution of mitochondrial oxphos complex proteins, or at 95°C, for resolution of β-actin and voltage-dependent anion channel (VDAC). Samples were then resolved on 12% SDS-PAGE gels and transferred to polyvinylidene difluoride membranes for immunoblotting. Total OXPHOS Rodent WB Antibody Cocktail (Abcam, ab110413), β-actin (Sigma-Aldrich, A5441), and VDAC (Cell Signaling Technology, 4661) were used to probe their respective targets. Horseradish peroxidase–conjugated secondary antibodies were anti-rabbit immunoglobulin G (IgG) (Millipore, AP132P) and anti-mouse IgG (Millipore, AP124P).

### Hargreaves test

Hindlimb withdrawal reaction to escalating heat stimuli was measured in conscious, unrestrained mice, as described elsewhere (*72*). Briefly, mice were allowed to acclimate to an observation chamber for 30 min before testing. Paw response latency to surface heat escalating at a rate of 1°C/s from a starting surface temperature of 30°C was measured using a Hargreaves apparatus. Triplicate measurements were made at 5-min intervals, and the mean was used to average paw heat response latency.

### Von Frey test

Hindlimb withdrawal reaction to light touch was measured in conscious, unrestrained mice, as described elsewhere (*72*). Briefly, mice were allowed to acclimate to an observation chamber for 30 min before testing. Paw withdrawal to pressure applied at the plantar surface was measured using von Frey filaments applied sequentially in an up-down protocol as originally described for rats (*73*) and subsequently modified for use in mice (*72*). Filaments were applied for 1 s at 1-s intervals with 5-min break between each set of stimulations, with five stimulations per filament. Response frequency for each filament was recorded, and 50% threshold was calculated using Hill equation (Origin 2017, OriginLab).

### NCV measurement

Conduction velocity of large myelinated motor fibers was measured in the sciatic nerve as described elsewhere (*72*). Briefly, mice were anesthetized with isoflurane, core and nerve temperatures were held at 37°C using heating pads and lamps, and fine needle stimulating electrodes were placed at the sciatic notch and Achilles tendon. Recording electrodes were inserted into the interosseus muscles of the ipsilateral paw. The sciatic nerve was stimulated using a PowerLab 4/30 to achieve maximal M wave amplitude (0.2 to 1.0 mV, 0.05-ms square waves), and the resulting electromyogram was stored to a computer running LabChart Pro (AD Instruments). NCV was calculated as the distance between stimulation sites divided by the latency of M wave peaks produced by stimulation at the two sites. The median of three separate measurements was used to represent NCV for each animal.

### RNA isolation and quantitative reverse transcription polymerase chain reaction

Total RNA was purified from frozen liver using the RNeasy Mini Kit (Qiagen) according to the manufacturer’s instructions. Complementary DNA (cDNA) was synthesized from 1 μg of total RNA using iScript Reverse Transcription Supermix for reverse transcription polymerase chain reaction (RT-PCR) (Bio-Rad Laboratories) according to the manufacturer’s instructions. Individual 10-μl SYBR Green real-time PCRs consisted of 2 μl of diluted cDNA, 5 μl of SYBR Green Supermix (Bio-Rad), and 1 μl of each 5 μM forward and reverse primers. For standardization of quantification, β-actin was amplified simultaneously. The PCR was carried out on 96-well plates on the CFX Connect Real-Time System (Bio-Rad CFX Manager v.3.1) using a three-stage program provided by the manufacturer: 95°C for 3 min, 40 cycles of 95°C for 10 s and 60°C for 30 s. Gene-specific primers used are listed in table S8.

### Quantification and statistical analysis

All results are depicted as means ± SEM of at least five biological replicates, as indicated in the figure legends, using GraphPad Prism (v.8.0.1). Three-dimensional plot of amino acids was produced using OriginPro (version 2021) (OriginLab Corporation). *P* values were calculated using two-way analysis of variance (ANOVA) or two-sided Student’s *t* test for each comparison either with or without adjustment for multiple comparisons. Data are means ± SEM of *n* = 5 to 8 animals per group. *P < 0.05, **P < 0.01, ***P < 0.001, and ****P < 0.0001.

## References

[1] J. B. Spinelli, M. C. Haigis, The multifaceted contributions of mitochondria to cellular metabolism. Nat. Cell Biol. 20, 745–754 (2018). 29950572 10.1038/s41556-018-0124-1 PMC6541229

[2] F. M. Yakes, B. Van Houten, Mitochondrial DNA damage is more extensive and persists longer than nuclear DNA damage in human cells following oxidative stress. Proc. Natl. Acad. Sci. U.S.A. 94, 514–519 (1997). 9012815 10.1073/pnas.94.2.514 PMC19544

[3] J. A. Stuart, M. F. Brown, Mitochondrial DNA maintenance and bioenergetics. Biochim. Biophys. Acta. Bioenerg. 1757, 79–89 (2006). 10.1016/j.bbabio.2006.01.003 16473322

[4] A. A. Johnson, K. A. Johnson, Exonuclease proofreading by human mitochondrial DNA polymerase. J. Biol. Chem. 276, 38097–38107 (2001). 11477094 10.1074/jbc.M106046200

[5] K.-S. Park, K.-J. Nam, J.-W. Kim, Y.-B. Lee, C.-Y. Han, J.-K. Jeong, H.-K. Lee, Y. K. Pak, Depletion of mitochondrial DNA alters glucose metabolism in SK-Hep1 cells. Am. J. Physiol. Metab. 280, E1007–E1014 (2001). 10.1152/ajpendo.2001.280.6.E1007 11350783

[6] X. R. Bao, S. E. Ong, O. Goldberger, J. Peng, R. Sharma, D. A. Thompson, S. B. Vafai, A. G. Cox, E. Marutani, F. Ichinose, W. Goessling, A. Regev, S. A. Carr, C. B. Clish, V. K. Mootha, Mitochondrial dysfunction remodels one-carbon metabolism in human cells. Elife 5, e10575 (2016). 27307216 10.7554/eLife.10575 PMC4911214

[7] A. Trifunovic, A. Wredenberg, M. Falkenberg, J. N. Spelbrink, A. T. Rovio, C. E. Bruder, M. Bohlooly-Y, S. Gldlöf, A. Oldfors, R. Wibom, J. Törnell, H. T. Jacobs, N. G. Larsson, Premature ageing in mice expressing defective mitochondrial DNA polymerase. Nature 429, 417–423 (2004). 15164064 10.1038/nature02517

[8] C. C. Kujoth, A. Hiona, T. D. Pugh, S. Someya, K. Panzer, S. E. Wohlgemuth, T. Hofer, A. Y. Seo, R. Sullivan, W. A. Jobling, J. D. Morrow, H. Van Remmen, J. M. Sedivy, T. Yamasoba, M. Tanokura, R. Weindruch, C. Leeuwenburgh, T. A. Prolla, Mitochondrial DNA mutations, oxidative stress, and apoptosis in mammalian aging. Science 309, 481–484 (2005). 16020738 10.1126/science.1112125

[9] M. Vermulst, J. Wanagat, G. C. Kujoth, J. H. Bielas, P. S. Rabinovitch, T. A. Prolla, L. A. Loeb, DNA deletions and clonal mutations drive premature aging in mitochondrial mutator mice. Nat. Genet. 40, 392–394 (2008). 18311139 10.1038/ng.95

[10] Y. Dai, T. Kiselak, J. Clark, E. Clore, K. Zheng, A. Cheng, G. C. Kujoth, T. A. Prolla, E. Maratos-Flier, D. K. Simon, Behavioral and metabolic characterization of heterozygous and homozygous POLG mutator mice. Mitochondrion 13, 282–291 (2013). 23542163 10.1016/j.mito.2013.03.006 PMC3682692

[11] D. F. Dai, T. Chen, J. Wanagat, M. Laflamme, D. J. Marcinek, M. J. Emond, C. P. Ngo, T. A. Prolla, P. S. Rabinovitch, Age-dependent cardiomyopathy in mitochondrial mutator mice is attenuated by overexpression of catalase targeted to mitochondria. Aging Cell 9, 536–544 (2010). 20456298 10.1111/j.1474-9726.2010.00581.x PMC3265170

[12] A.-M. Joseph, P. J. Adhihetty, N. R. Wawrzyniak, S. E. Wohlgemuth, A. Picca, G. C. Kujoth, T. A. Prolla, C. Leeuwenburgh, Dysregulation of mitochondrial quality control processes contribute to sarcopenia in a mouse model of premature aging. PLOS ONE 8, e69327 (2013). 23935986 10.1371/journal.pone.0069327 PMC3720551

[13] J. D. Stumpf, R. P. Saneto, W. C. Copeland, Clinical and molecular features of polg-related mitochondrial disease. Cold Spring Harb. Perspect. Biol. 5, a011395 (2013). 23545419 10.1101/cshperspect.a011395 PMC3683902

[14] A. Penno, M. M. Reilly, H. Houlden, M. Laurá, K. Rentsch, V. Niederkofler, E. T. Stoeckli, G. Nicholson, F. Eichler, R. H. Brown Jr., A. Von Eckardstein, T. Hornemann, Hereditary sensory neuropathy type 1 is caused by the accumulation of two neurotoxic sphingolipids. J. Biol. Chem. 285, 11178–11187 (2010). 20097765 10.1074/jbc.M109.092973 PMC2856995

[15] A. Othman, C. H. Saely, A. Muendlein, A. Vonbank, H. Drexel, A. von Eckardstein, T. Hornemann, Plasma 1-deoxysphingolipids are predictive biomarkers for type 2 diabetes mellitus. BMJ Open Diabetes Res. Care 3, e000073 (2015). 10.1136/bmjdrc-2014-000073 PMC4368929 25815206

[16] V. Fridman, S. Zarini, S. Sillau, K. Harrison, B. C. Bergman, E. L. Feldman, J. E. B. Reusch, B. C. Callaghan, Altered plasma serine and 1-deoxydihydroceramide profiles are associated with diabetic neuropathy in type 2 diabetes and obesity. J. Diabetes Complications 35, 107852 (2021). 33485750 10.1016/j.jdiacomp.2021.107852 PMC8114795

[17] R. Kramer, J. Bielawski, E. Kistner-Griffin, A. Othman, I. Alecu, D. Ernst, D. Kornhauser, T. Hornemann, S. Spassieva, Neurotoxic 1-deoxysphingolipids and paclitaxel-induced peripheral neuropathy. FASEB J. 29, 4461–4472 (2015). 26198449 10.1096/fj.15-272567 PMC4608911

[18] Mitochondrial Medicine Society’s Committee on Diagnosis, R. H. Haas, S. Parikh, M. J. Falk, R. P. Saneto, N. I. Wolf, N. Darin, L.-J. Wong, B. H. Cohen, R. K. Naviaux, The in-depth evaluation of suspected mitochondrial disease. Mol. Genet. Metab. 94, 16–37 (2008). 18243024 10.1016/j.ymgme.2007.11.018 PMC2810849

[19] J. M. Phang, *Current Topics in Cellular Regulation* (Academic Press, 1985), vol. 25, pp. 91–132. 10.1016/b978-0-12-152825-6.50008-4 2410198

[20] J. Zhu, S. Schwörer, M. Berisa, Y. J. Kyung, K. W. Ryu, J. Yi, X. Jiang, J. R. Cross, C. B. Thompson, Mitochondrial NADP(H) generation is essential for proline biosynthesis. Science 372, eabd5491 (2021). 10.1126/science.abd5491 PMC8241437 33888598

[21] D. H. Tran, R. Kesavan, H. Rion, M. H. Soflaee, A. Solmonson, D. Bezwada, H. S. Vu, F. Cai, J. A. Phillips III, R. J. DeBerardinis, G. Hoxhaj, Mitochondrial NADP^+^ is essential for proline biosynthesis during cell growth. Nat. Metab. 3, 571–585 (2021). 33833463 10.1038/s42255-021-00374-y PMC9210447

[22] F. F. Diehl, C. A. Lewis, B. P. Fiske, M. G. Vander Heiden, Cellular redox state constrains serine synthesis and nucleotide production to impact cell proliferation. Nat. Metab. 1, 861–867 (2019). 31598584 10.1038/s42255-019-0108-x PMC6785045

[23] A. S. Tibbetts, D. R. Appling, Compartmentalization of mammalian folate-mediated one-carbon metabolism. Annu. Rev. Nutr. 30, 57–81 (2010). 20645850 10.1146/annurev.nutr.012809.104810

[24] M. Wallace, C. R. Green, L. S. Roberts, Y. M. Lee, J. L. McCarville, J. Sanchez-Gurmaches, N. Meurs, J. M. Gengatharan, J. D. Hover, S. A. Phillips, T. P. Ciaraldi, D. A. Guertin, P. Cabrales, J. S. Ayres, D. K. Nomura, R. Loomba, C. M. Metallo, Enzyme promiscuity drives branched-chain fatty acid synthesis in adipose tissues. Nat. Chem. Biol. 14, 1021–1031 (2018). 30327559 10.1038/s41589-018-0132-2 PMC6245668

[25] C. J. Lynch, B. J. Patson, J. Anthony, A. Vaval, L. S. Jefferson, T. C. Vary, Leucine is a direct-acting nutrient signal that regulates protein synthesis in adipose tissue. Am. J. Physiol. Endocrinol. Metab. 283, E503–E513 (2002). 12169444 10.1152/ajpendo.00084.2002

[26] P. M. Quirós, M. A. Prado, N. Zamboni, D. D’Amico, R. W. Williams, D. Finley, S. P. Gygi, J. Auwerx, Multi-omics analysis identifies ATF4 as a key regulator of the mitochondrial stress response in mammals. J. Cell Biol. 216, 2027–2045 (2017). 28566324 10.1083/jcb.201702058 PMC5496626

[27] L. B. Sullivan, D. Y. Gui, A. M. Hosios, L. N. Bush, E. Freinkman, M. G. Vander Heiden, Supporting aspartate biosynthesis is an essential function of respiration in proliferating cells. Cell 162, 552–563 (2015). 26232225 10.1016/j.cell.2015.07.017 PMC4522278

[28] K. Birsoy, T. Wang, W. W. Chen, E. Freinkman, M. Abu-Remaileh, D. M. Sabatini, An essential role of the mitochondrial electron transport chain in cell proliferation is to enable aspartate synthesis. Cell 162, 540–551 (2015). 26232224 10.1016/j.cell.2015.07.016 PMC4522279

[29] D. N. Hauser, A. A. Dillman, J. Ding, Y. Li, M. R. Cookson, Post-translational decrease in respiratory chain proteins in the polg mutator mouse brain. PLOS ONE 9, e94646 (2014). 24722488 10.1371/journal.pone.0094646 PMC3983222

[30] K. L. McLaughlin, K. A. Kew, J. M. McClung, K. H. Fisher-Wellman, Subcellular proteomics combined with bioenergetic phenotyping reveals protein biomarkers of respiratory insufficiency in the setting of proofreading-deficient mitochondrial polymerase. Sci. Rep. 10, 3603 (2020). 32107436 10.1038/s41598-020-60536-y PMC7046634

[31] J. M. Ross, G. Coppotelli, R. M. Branca, K. M. Kim, J. Lehtiö, D. A. Sinclair, L. Olson, Voluntary exercise normalizes the proteomic landscape in muscle and brain and improves the phenotype of progeroid mice. Aging Cell 18, e13029 (2019). 31489782 10.1111/acel.13029 PMC6826127

[32] E. Scalais, B. Francois, P. Schlesser, R. Stevens, C. Nuttin, J. J. Martin, R. Van Coster, S. Seneca, F. Roels, G. Van Goethem, A. Löfgren, L. De Meirleir, Polymerase gamma deficiency (POLG): Clinical course in a child with a two stage evolution from infantile myocerebrohepatopathy spectrum to an Alpers syndrome and neuropathological findings of Leigh’s encephalopathy. Eur. J. Paediatr. Neurol. 16, 542–548 (2012). 22342071 10.1016/j.ejpn.2012.01.013

[33] H. Montassir, Y. Maegaki, K. Murayama, T. Yamazaki, M. Kohda, A. Ohtake, H. Iwasa, Y. Yatsuka, Y. Okazaki, C. Sugiura, I. Nagata, M. Toyoshima, Y. Saito, M. Itoh, I. Nishino, K. Ohno, Myocerebrohepatopathy spectrum disorder due to POLG mutations: A clinicopathological report. Brain Dev. 37, 719–724 (2015). 25466440 10.1016/j.braindev.2014.10.013

[34] O. Hikmat, C. Tzoulis, W. K. Chong, L. Chentouf, C. Klingenberg, C. Fratter, L. J. Carr, P. Prabhakar, N. Kumaraguru, P. Gissen, J. H. Cross, T. S. Jacques, J. W. Taanman, L. A. Bindoff, S. Rahman, The clinical spectrum and natural history of early-onset diseases due to DNA polymerase gamma mutations. Genet. Med. 19, 1217–1225 (2017). 28471437 10.1038/gim.2017.35

[35] B. N. Harding, Progressive neuronal degeneration of childhood with liver disease (Alpers-Huttenlocher Syndrome): A personal review. J. Child Neurol. 5, 273–287 (1990). 2246481 10.1177/088307389000500402

[36] C. E. Wall, J. Whyte, J. M. Suh, W. Fan, B. Collins, C. Liddle, R. T. Yu, A. R. Atkins, J. C. Naviaux, K. Li, A. T. Bright, W. A. Alaynick, M. Downes, R. K. Naviaux, R. M. Evans, High-fat diet and FGF21 cooperatively promote aerobic thermogenesis in mtDNA mutator mice. Proc. Natl. Acad. Sci. U.S.A. 112, 8714–8719 (2015). 26124126 10.1073/pnas.1509930112 PMC4507233

[37] R. G. Fox, S. Magness, G. C. Kujoth, T. A. Prolla, N. Maeda, Mitochondrial DNA polymerase editing mutation, PolgD257A, disturbs stem-progenitor cell cycling in the small intestine and restricts excess fat absorption. Am. J. Physiol. Liver Physiol. 302, G914–G924 (2012). 10.1152/ajpgi.00402.2011 PMC3362078 22345551

[38] J. Zborowski, A. Dygas, L. Wojtczak, Phosphatidylserine decarboxylase is located on the external side of the inner mitochondrial membrane. FEBS Lett. 157, 179–182 (1983). 6862014 10.1016/0014-5793(83)81141-7

[39] E. P. Kennedy, S. B. Weiss, The function of cytidine coenzymes in the biosynthesis of phospholipides. J. Biol. Chem. 222, 193–214 (1956). 13366993

[40] D. E. Vance, Phospholipid methylation in mammals: From biochemistry to physiological function. Biochim. Biophys. Acta 1838, 1477–1487 (2014). 24184426 10.1016/j.bbamem.2013.10.018

[41] J. Duan, A. H. Merrill Jr., 1-deoxysphingolipids encountered exogenously and made de novo: Dangerous mysteries inside an enigma. J. Biol. Chem. 290, 15380–15389 (2015). 25947379 10.1074/jbc.R115.658823 PMC4505451

[42] M. A. Lone, T. Santos, I. Alecu, L. C. Silva, T. Hornemann, 1-Deoxysphingolipids. Biochim. Biophys. Acta Mol. Cell Biol. Lipids 1864, 512–521 (2019). 30625374 10.1016/j.bbalip.2018.12.013

[43] F. S. Eichler, T. Hornemann, A. McCampbell, D. Kuljis, A. Penno, D. Vardeh, E. Tamrazian, K. Garofalo, H. J. Lee, L. Kini, M. Selig, M. Frosch, K. Gable, A. Von Eckardstein, C. J. Woolf, G. Guan, J. M. Harmon, T. M. Dunn, R. H. Brown, Overexpression of the wild-type SPT1 subunit lowers desoxysphingolipid levels and rescues the phenotype of HSAN1. J. Neurosci. 29, 14646–14651 (2009). 19923297 10.1523/JNEUROSCI.2536-09.2009 PMC3849752

[44] M. L. Gantner, K. Eade, M. Wallace, M. K. Handzlik, R. Fallon, J. Trombley, R. Bonelli, S. Giles, S. Harkins-Perry, T. F. C. Heeren, L. Sauer, Y. Ideguchi, M. Baldini, L. Scheppke, M. I. Dorrell, M. Kitano, B. J. Hart, C. Cai, T. Nagasaki, M. G. Badur, M. Okada, S. M. Woods, C. Egan, M. Gillies, R. Guymer, F. Eichler, M. Bahlo, M. Fruttiger, R. Allikmets, P. S. Bernstein, C. M. Metallo, M. Friedlander, Serine and lipid metabolism in macular disease and peripheral neuropathy. N. Engl. J. Med. 381, 1422–1433 (2019). 31509666 10.1056/NEJMoa1815111 PMC7685488

[45] T. Muthusamy, T. Cordes, M. K. Handzlik, L. You, E. W. Lim, J. Gengatharan, A. F. M. Pinto, M. G. Badur, M. J. Kolar, M. Wallace, A. Saghatelian, C. M. Metallo, Serine restriction alters sphingolipid diversity to constrain tumour growth. Nature 586, 790–795 (2020). 32788725 10.1038/s41586-020-2609-x PMC7606299

[46] A. Ando, M. Oka, Y. Satomi, Deoxysphingolipids and ether-linked diacylglycerols accumulate in the tissues of aged mice. Cell Biosci. 9, 61 (2019). 31402974 10.1186/s13578-019-0324-9 PMC6683348

[47] D. Pareyson, G. Piscosquito, I. Moroni, E. Salsano, M. Zeviani, Peripheral neuropathy in mitochondrial disorders. Lancet Neurol. 12, 1011–1024 (2013). 24050734 10.1016/S1474-4422(13)70158-3

[48] R. Z. Hill, T. Morita, R. B. Brem, D. M. Bautista, S1PR3 mediates itch and pain via distinct TRP channel-dependent pathways. J. Neurosci. 38, 7833–7843 (2018). 30082422 10.1523/JNEUROSCI.1266-18.2018 PMC6125817

[49] C. Clarke, R. Xiao, E. Place, Z. Zhang, N. Sondheimer, M. Bennett, M. Yudkoff, M. J. Falk, Mitochondrial respiratory chain disease discrimination by retrospective cohort analysis of blood metabolites. Mol. Genet. Metab. 110, 145–152 (2013). 23920046 10.1016/j.ymgme.2013.07.011 PMC3812452

[50] N. Esteitie, R. Hinttala, R. Wibom, H. Nilsson, N. Hance, K. Naess, K. Teär-Fahnehjelm, U. Von Döbeln, K. Majamaa, N.-G. Larsson, Secondary metabolic effects in complex I deficiency. Ann. Neurol. 58, 544–552 (2005). 16044424 10.1002/ana.20570

[51] J. T. Legault, L. Strittmatter, J. Tardif, R. Sharma, V. Tremblay-Vaillancourt, C. Aubut, G. Boucher, C. B. Clish, D. Cyr, C. Daneault, P. J. Waters; LSFC Consortium, C. Morin, C. Laprise, J. D. Rioux, V. K. Mootha, C. D. Rosiers, A metabolic signature of mitochondrial dysfunction revealed through a monogenic form of leigh syndrome. Cell Rep. 13, 981–989 (2015). 26565911 10.1016/j.celrep.2015.09.054 PMC4644511

[52] A. Saleem, A. Safdar, Y. Kitaoka, X. Ma, O. S. Marquez, M. Akhtar, A. Nazli, R. Suri, J. Turnbull, M. A. Tarnopolsky, Polymerase gamma mutator mice rely on increased glycolytic flux for energy production. Mitochondrion 21, 19–26 (2015). 25575636 10.1016/j.mito.2014.12.001

[53] M. J. Roef, S. C. Kalhan, D.-J. Reijngoud, K. De Meer, R. Berger, Lactate disposal via gluconeogenesis is increased during exercise in patients with mitochondrial myopathy due to complex I deficiency. Pediatr. Res. 51, 592–597 (2002). 11978882 10.1203/00006450-200205000-00008

[54] A. W. El-Hattab, J. W. Hsu, L. T. Emrick, L. J. C. Wong, W. J. Craigen, F. Jahoor, F. Scaglia, Restoration of impaired nitric oxide production in MELAS syndrome with citrulline and arginine supplementation. Mol. Genet. Metab. 105, 607–614 (2012). 22325939 10.1016/j.ymgme.2012.01.016 PMC4093801

[55] E. Ansó, S. E. Weinberg, L. P. Diebold, B. J. Thompson, S. Malinge, P. T. Schumacker, X. Liu, Y. Zhang, Z. Shao, M. Steadman, K. M. Marsh, J. Xu, J. D. Crispino, N. S. Chandel, The mitochondrial respiratory chain is essential for haematopoietic stem cell function. Nat. Cell Biol. 19, 614–625 (2017). 28504706 10.1038/ncb3529 PMC5474760

[56] S. E. Weinberg, B. D. Singer, E. M. Steinert, C. A. Martinez, M. M. Mehta, I. Martínez-Reyes, P. Gao, K. A. Helmin, H. Abdala-Valencia, L. A. Sena, P. T. Schumacker, L. A. Turka, N. S. Chandel, Mitochondrial complex III is essential for suppressive function of regulatory T cells. Nature 565, 495–499 (2019). 30626970 10.1038/s41586-018-0846-z PMC6345596

[57] A. M. Intlekofer, R. G. Dematteo, S. Venneti, L. W. S. Finley, C. Lu, A. R. Judkins, A. S. Rustenburg, P. B. Grinaway, J. D. Chodera, J. R. Cross, C. B. Thompson, Hypoxia induces production of L-2-hydroxyglutarate. Cell Metab. 22, 304–311 (2015). 26212717 10.1016/j.cmet.2015.06.023 PMC4527873

[58] P. Felig, The glucose-alanine cycle. Metabolism 22, 179–207 (1973). 4567003 10.1016/0026-0495(73)90269-2

[59] C. C. Tseng, C. J. Chen, J. H. Yen, H. Y. Huang, J. G. Chang, S. J. Chang, W. T. Liao, Next-generation sequencing profiling of mitochondrial genomes in gout. Arthritis Res. Ther. 20, 137 (2018). 29976239 10.1186/s13075-018-1637-5 PMC6034246

[60] S. Herzig, R. J. Shaw, AMPK: Guardian of metabolism and mitochondrial homeostasis. Nat. Rev. Mol. Cell Biol. 19, 121–135 (2018). 28974774 10.1038/nrm.2017.95 PMC5780224

[61] C. Ren, J. Liu, J. Zhou, H. Liang, Y. Zhu, Q. Wang, Y. Leng, Z. Zhang, Y. Yuan, Z. Wang, Y. Yin, Lipidomic profiling of plasma samples from patients with mitochondrial disease. Biochem. Biophys. Res. Commun. 500, 124–131 (2018). 29627572 10.1016/j.bbrc.2018.03.160

[62] C. R. Ferreira, S. M. I. Goorden, A. Soldatos, H. M. Byers, J. M. M. Ghauharali-van der Vlugt, F. S. Beers-Stet, C. Groden, C. D. van Karnebeek, W. A. Gahl, F. M. Vaz, X. Jiang, H. J. Vernon, Deoxysphingolipid precursors indicate abnormal sphingolipid metabolism in individuals with primary and secondary disturbances of serine availability. Mol. Genet. Metab. 124, 204–209 (2018). 29789193 10.1016/j.ymgme.2018.05.001 PMC6057808

[63] N. U. Schwartz, I. Mileva, M. Gurevich, J. Snider, Y. A. Hannun, L. M. Obeid, Quantifying 1-deoxydihydroceramides and 1-deoxyceramides in mouse nervous system tissue. Prostaglandins Other Lipid Mediat. 141, 40–48 (2019). 30790665 10.1016/j.prostaglandins.2019.02.005 PMC6467697

[64] I. Alecu, A. Tedeschi, N. Behler, K. Wunderling, C. Lamberz, M. A. R. Lauterbach, A. Gaebler, D. Ernst, P. P. Van Veldhoven, A. Al-Amoudi, E. Latz, A. Othman, L. Kuerschner, T. Hornemann, F. Bradke, C. Thiele, A. A. Penno, Localization of 1-deoxysphingolipids to mitochondria induces mitochondrial dysfunction. J. Lipid Res. 58, 42–59 (2017). 27881717 10.1194/jlr.M068676 PMC5234710

[65] A. G. Haribowo, J. Thomas Hannich, A. H. Michel, M. Megyeri, M. Schuldiner, B. Kornmann, H. Riezman, Cytotoxicity of 1-deoxysphingolipid unraveled by genome-wide genetic screens and lipidomics in *Saccharomyces cerevisiae*. Mol. Biol. Cell 30, 2814–2826 (2019). 31509475 10.1091/mbc.E19-07-0364 PMC6789163

[66] T. Cordes, C. M. Metallo, *Methods in Molecular Biology* (Humana Press Inc., 2019), vol. 1978, pp. 219–241. 10.1007/978-1-4939-9236-2_14 31119666

[67] C. A. Fernandez, C. Des Rosiers, S. F. Previs, F. David, H. Brunengraber, Correction of ^13^C mass isotopomer distributions for natural stable isotope abundance. J. Mass Spectrom. 31, 255–262 (1996). 8799277 10.1002/(SICI)1096-9888(199603)31:3<255::AID-JMS290>3.0.CO;2-3

[68] M. R. Antoniewicz, J. K. Kelleher, G. Stephanopoulos, Measuring deuterium enrichment of glucose hydrogen atoms by gas chromatography/mass spectrometry. Anal. Chem. 83, 3211–3216 (2011). 21413777 10.1021/ac200012p PMC3562696

[69] G. M. MacKay, L. Zheng, N. J. F. Van Den Broek, E. Gottlieb, *Methods in Enzymology* (Academic Press Inc., 2015), vol. 561, pp. 171–196. 10.1016/bs.mie.2015.05.016 26358905

[70] J. Bielawski, J. S. Pierce, J. Snider, B. Rembiesa, Z. M. Szulc, A. Bielawska, Comprehensive quantitative analysis of bioactive sphingolipids by high-performance liquid chromatography–tandem mass spectrometry. Methods Mol. Biol. 579, 443–467 (2009). 19763489 10.1007/978-1-60761-322-0_22

[71] R. R. Wolfe, D. L. Chinkes, *Isotope Tracers in Metabolic Research: Principles and Practice of Kinetic Analysis* (John Wiley & Sons Inc., ed. 2, 2004).

[72] C. G. Jolivalt, K. E. Frizzi, L. Guernsey, A. Marquez, J. Ochoa, M. Rodriguez, N. A. Calcutt, Peripheral neuropathy in mouse models of diabetes. Curr. Protoc. Mouse Biol. 6, 223–255 (2016). 27584552 10.1002/cpmo.11 PMC5023323

[73] S. R. Chaplan, F. W. Bach, J. W. Pogrel, J. M. Chung, T. L. Yaksh, Quantitative assessment of tactile allodynia in the rat paw. J. Neurosci. Methods 53, 55–63 (1994). 7990513 10.1016/0165-0270(94)90144-9

[74] G. Karsai, M. Lone, Z. Kutalik, J. Thomas Brenna, H. Li, D. Pan, A. von Eckardstein, X. Thorsten Hornemann, FADS3 is a Δ14Z sphingoid base desaturase that contributes to gender differences in the human plasma sphingolipidome. J. Biol. Chem. 295, 1889–1897 (2020). 31862735 10.1074/jbc.AC119.011883 PMC7029104

