# Supplementary material for: Progressive alterations in amino acid and lipid metabolism correlate with peripheral neuropathy in PolgD257A mice
Source: Sci Adv. 2021 Oct 15;7(42):eabj4077. doi: 10.1126/sciadv.abj4077 (PMC8519573; doi:10.1126/sciadv.abj4077)
Supplement: Supplementary file 1 — Figs. S1 to S6 Legends for tables S1 to S5 Tables S6 to S8 [file sciadv.abj4077_sm.pdf]

Science Advances
AAAS

Supplementary Materials for

Progressive alterations in amino acid and lipid metabolism correlate with peripheral neuropathy in *Polg*^{D257A} mice

Esther W. Lim, Michal K. Handzlik, Elijah Trefts, Jivani M. Gengatharan, Carlos M. Pondevida, Reuben J. Shaw, Christian M. Metallo*

*Corresponding author. Email: metallo@salk.edu

Published 15 October 2021, *Sci. Adv.* **7**, eabj4077 (2021)
DOI: 10.1126/sciadv.abj4077

The PDF file includes:

Figs. S1 to S6
Legends for tables S1 to S5
Tables S6 to S8

Other Supplementary Material for this manuscript includes the following:

Tables S1 to S5

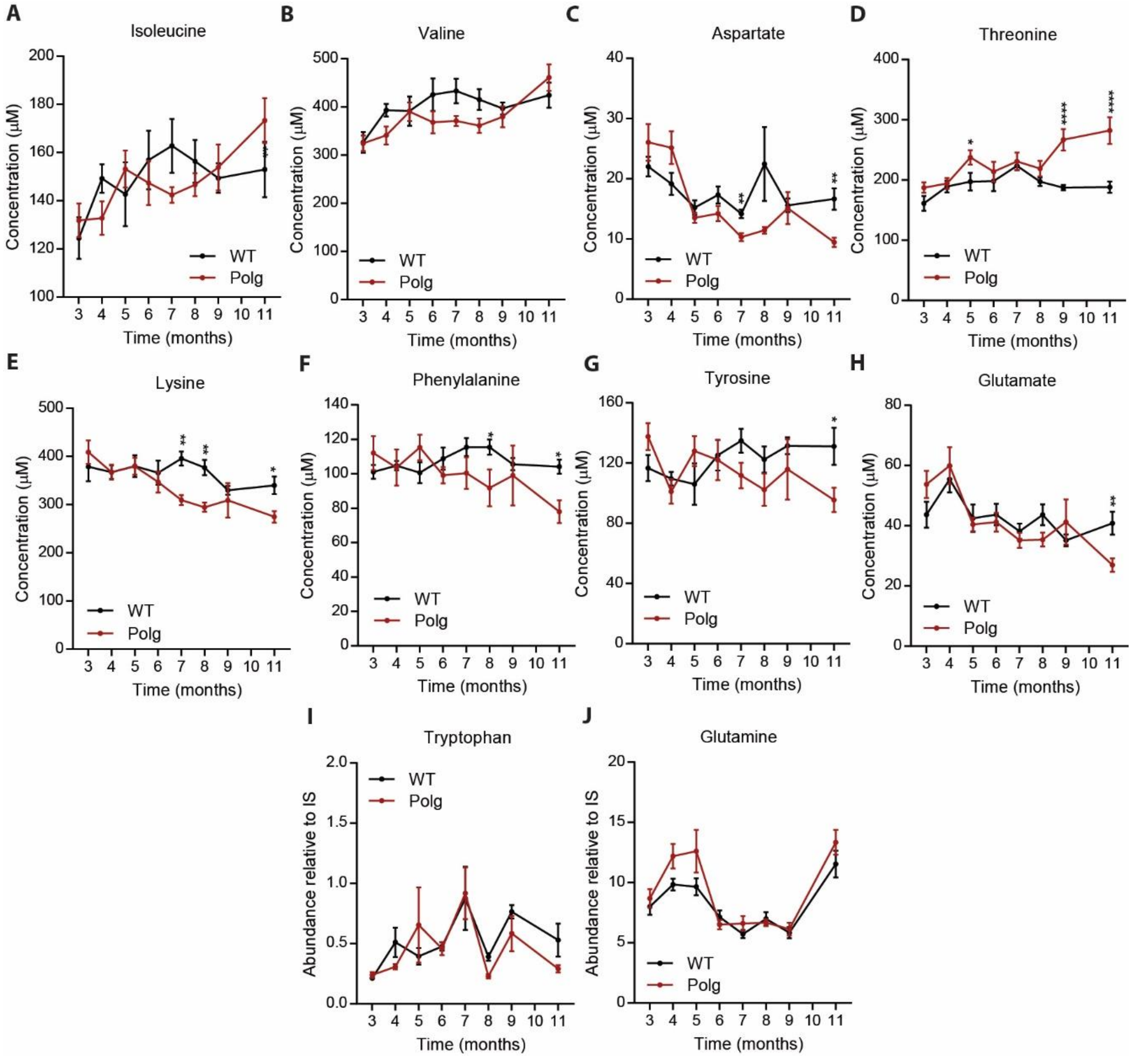

Fig. S1.

Amino acid profiles in *Polg* mice

Concentration of plasma isoleucine (**A**), valine (**B**), aspartate (**C**), threonine (**D**), lysine (**E**), phenylalanine (**F**), tyrosine (**G**), and glutamate (**H**) in WT and *Polg* mice from 3 to 11 months of age.
Abundance of plasma tryptophan (**I**) and glutamine (**J**) relative to internal standard from 3 to 11 months of age.

Two-way ANOVA for each comparison with no adjustment for multiple comparisons. Data are mean ± s.e.m. of n=7-8 animals per group. *P < 0.05, **P < 0.01, ***P < 0.001, ****P<0.0001.

A

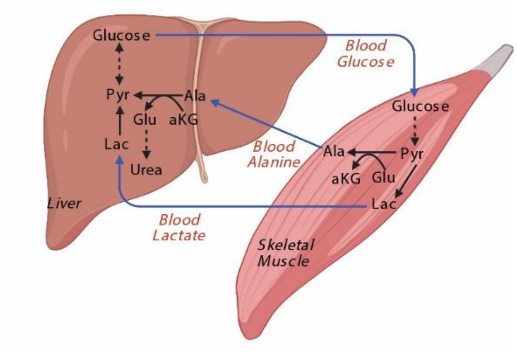

Fig. S2.

Metabolite cycling in *Polg* mice

(**A**) Schematic of Cori and Cahill cycling. Created with Biorender.com.

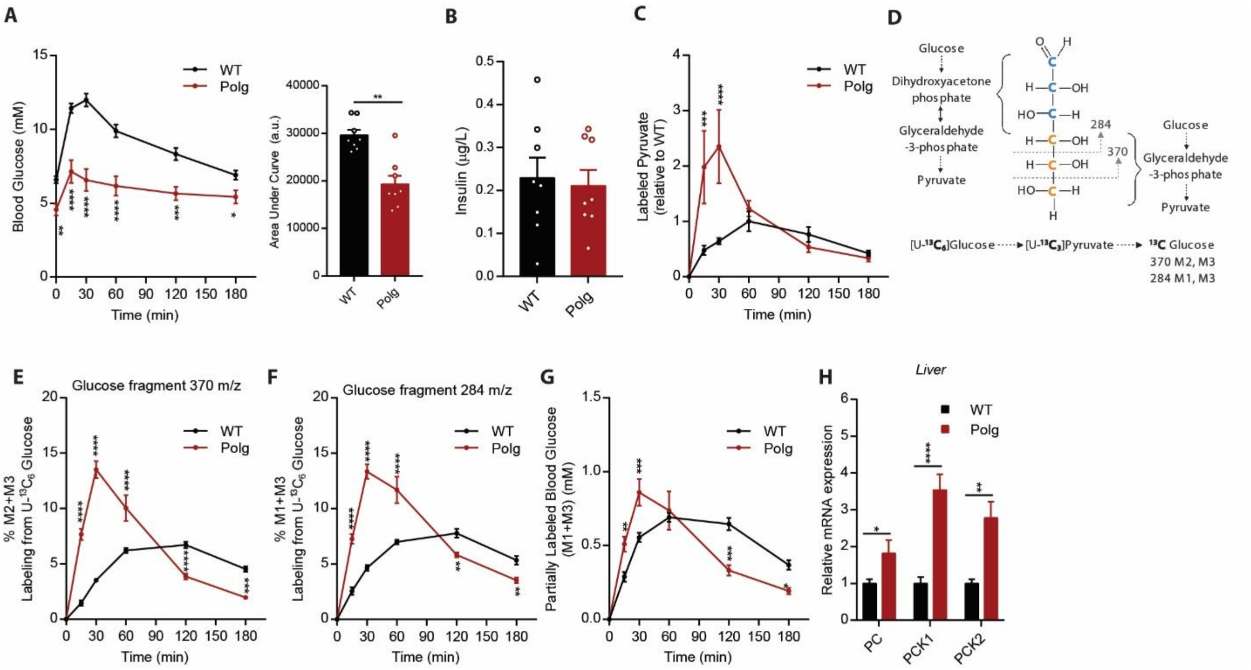

Fig. S3.

Differential glucose metabolism in *Polg* mice

(**A**) Concentration of blood glucose over time upon bolus administration of glucose via intraperitoneal injection and quantitation of area under the curve (a.u., arbitrary units).
(**B**) Concentration of fasting plasma insulin in WT and *Polg* mice.
(**C**) Levels of ^{13}C labeled pyruvate (1-M0 fraction multiplied with metabolite abundance) relative to internal standard in plasma over time. Values are normalized to WT, maximum WT value is set to 1.
(**D**) Schematic overview of positional information obtained from
GC-MS analysis of aldonitrile pentapropionate glucose fragments 284 and 370 m/z. Created with Biorender.com.
Fraction of M+2 and M+3 isotopomers of glucose fragment 370 m/z (**E**) and M+1 and M+3 isotopomers of glucose fragment 284 m/z (**F**) over time.
(**G**) Levels of partially labeled glucose (sum of M+1 and M+3 isotopomers of 284 m/z fragment) in plasma over time.
(**H**) Relative mRNA expression of pyruvate carboxylase (PC), phosphoenolpyruvate carboxykinase 1 (PCK1), and PCK2 in liver.

Two-sided Student's t-test for each comparison with no adjustment for multiple comparisons. Data are mean ± s.e.m. of n=7-8 animals per group. *P < 0.05, **P < 0.01, ***P < 0.001, ****P<0.0001.

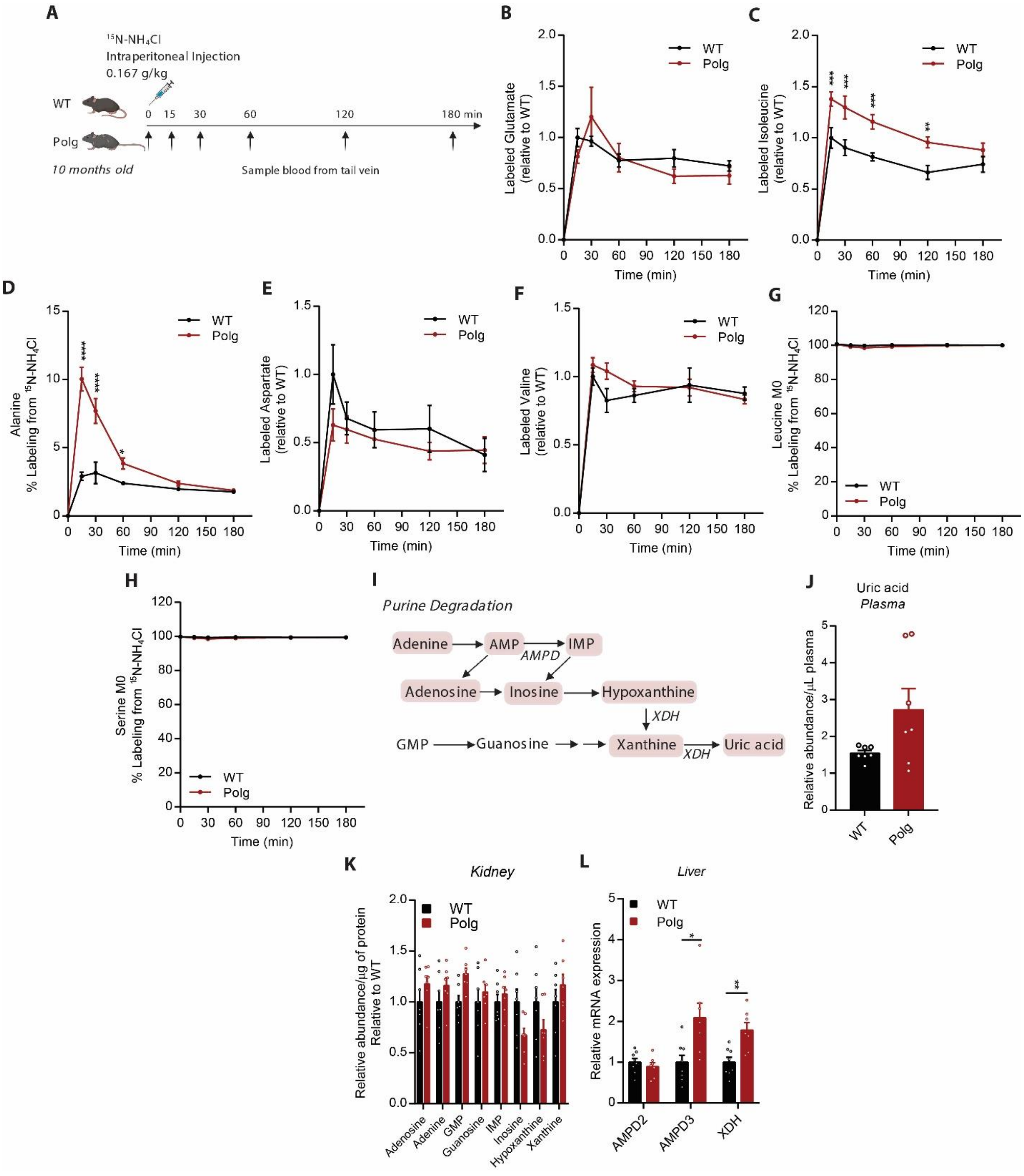

Fig. S4.

Nitrogen metabolism and ^{15}N-NH_4Cl tracing in *Polg* mice.

(**A**) Schematic of ^{15}N-labeled ammonium chloride (^{15}N-NH_4Cl) tracing experiment. Created with Biorender.com.

Levels of ^{15}N-labeled glutamate (**B**) and isoleucine (**C**) in plasma relative to internal standard over time. Values are normalized to WT, maximum WT value is set to 1.

(**D**) Fraction of ^{15}N labeling on plasma alanine over duration of experiment.

Levels of ^{15}N-labeled aspartate (**E**) and valine (**F**) in plasma relative to internal standard over time. Values are normalized to WT, maximum WT value is set to 1.
M0 enrichment of ^{15}N label on leucine (**G**) and serine (**H**) over time. 100% M0 labeling indicates no incorporation of ^{15}N label onto metabolite.
(**I**) Schematic of purine degradation pathway. Created with Biorender.com.
(**J**) Abundance of uric acid relative to internal standard in plasma at 12 months of age.
(**K**) Abundance of purine catabolism intermediates relative to internal standard in the kidney at 12 months of age. Abundances were normalized to µg protein per tissue.
(**L**) Relative mRNA expression of AMP deaminase 2 (AMPD2), AMPD3, and xanthine dehydrogenase (XDH) in liver.

Two-sided Student's t-test for each comparison with no adjustment for multiple comparisons. Data are mean ± s.e.m. of n=7-8 animals per group. *P < 0.05, **P < 0.01, ***P < 0.001, ****P<0.0001.

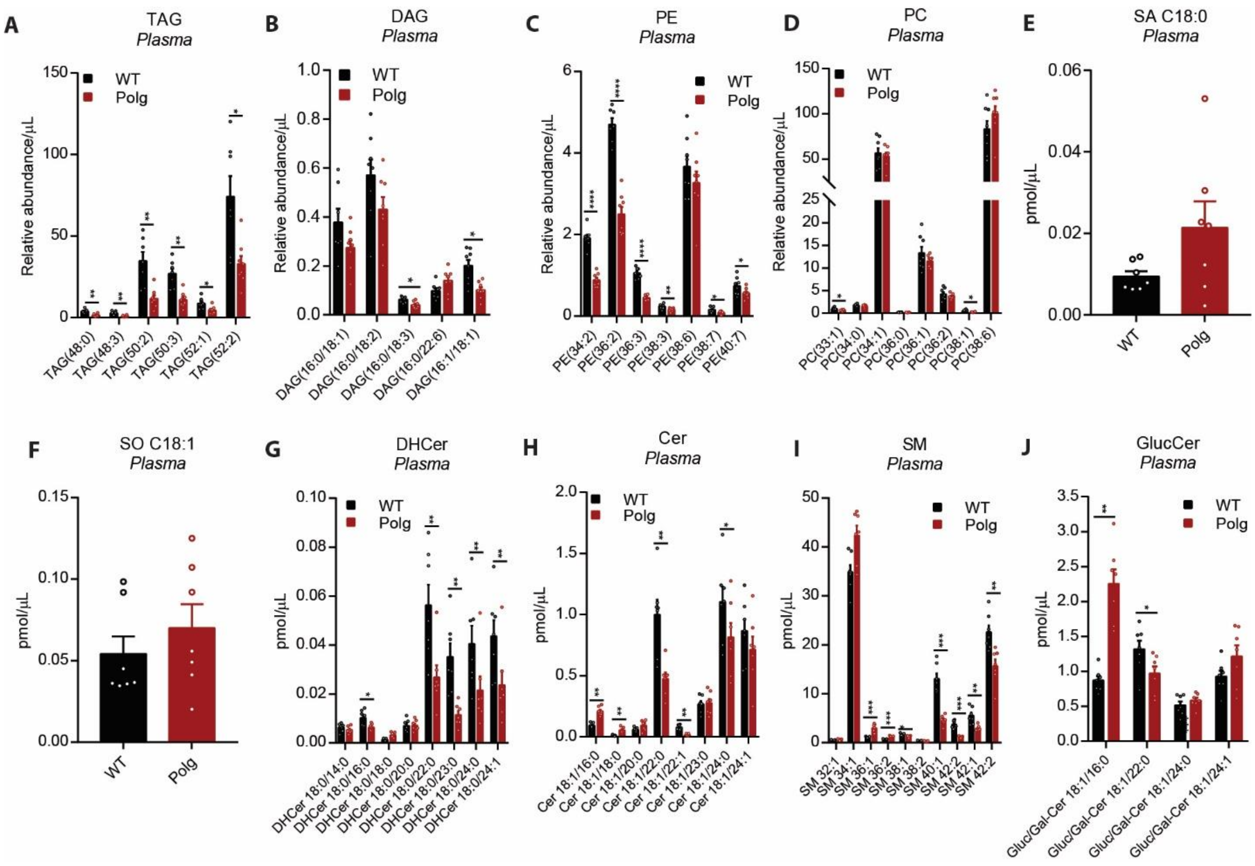

Fig. S5.

Lipid abundances in WT and *Polg* mice

Abundances of individual plasma triacylglycerols (TAGs) (**A**), diacylglycerols (DAGs) (**B**), phosphatidylethanolamine (**C**), and phosphatidylcholine (**D**) relative to internal standard per µL of plasma in WT and *Polg* mice.

Concentrations of plasma sphinganine (**E**) and sphingosine (**F**) in WT and *Polg* mice.

Concentration of individual dihydroceramides (DHCer) (**G**), ceramides (Cer) (**H**), sphingomyelin (SM) (**I**), and glucosylceramides (GlucCer) (**J**) in WT and *Polg* mice.

Two-sided Student's t-test for each comparison with no adjustment for multiple comparisons. Data are mean ± s.e.m. of n=7-8 animals per group. *P < 0.05, **P < 0.01, ***P < 0.001, ****P<0.0001.

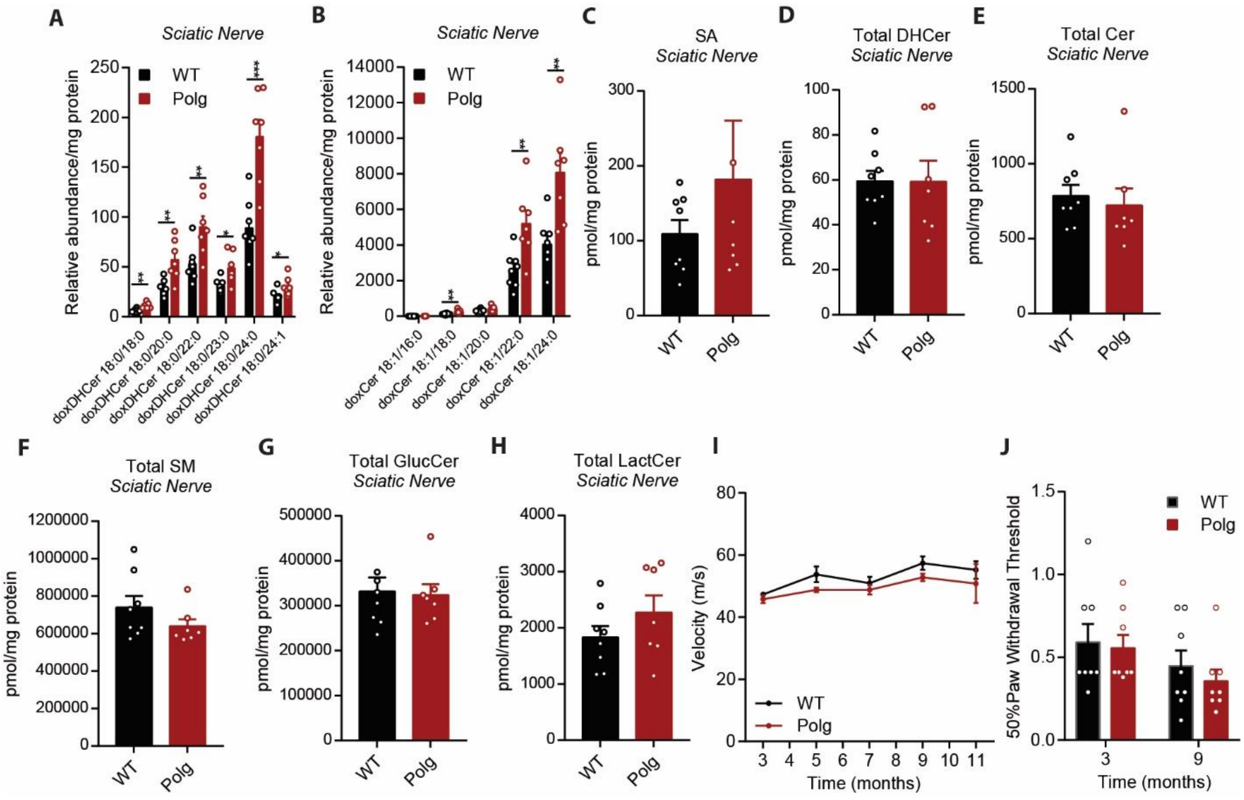

Fig. S6.

Sciatic nerve sphingolipids, nerve conduction velocity, and mechanical nociception in *Polg* mice

Abundances of individual sciatic nerve deoxydihydroceramides (doxDHCer) (**A**) and deoxyceramides (doxCer) (**B**) in WT and *Polg* mice.

Concentrations of total sciatic nerve sphinganine (SA) (**C**), dihydroceramide (DHCer) (**D**), ceramide (Cer) (**E**), sphingomyelin (SM) (**F**), glucosylceramides (GlucCer) (**G**), and lactosylceramides (LactCer) (**H**) in WT and *Polg* mice.

(**I**) Motor nerve conduction velocity measured every 2 months in WT and *Polg* mice.

(**J**) Paw response to von Frey filaments measured at 3 and 9 months in WT and *Polg* mice.

Two-sided Student's t-test for each comparison with no adjustment for multiple comparisons. Data are mean ± s.e.m. of n=7-8 animals per group. *P < 0.05, **P < 0.01, ***P < 0.001, ****P<0.0001.

Table S1.

Plasma amino acid concentration and fold-change (*Polg*/WT) as shown in Fig. 2 and Fig. S1

Provided as a separate file (.xlsx) due to size.

Table S2.
Tissue metabolite abundance and fold-change (*Polg*/WT) as shown in Fig. 3D

Provided as a separate file (.xlsx) due to size.

Table S3.

Tissue amino acid concentration and fold-change (*Polg*/WT) as shown in Fig. 3E

Provided as a separate file (.xlsx) due to size.

Table S4.
Tissue nucleotide phosphate abundance and fold-change (*Polg*/WT) as shown in Fig.4I

Provided as a separate file (.xlsx) due to size.

Table S5.

Tissue purine catabolism intermediate abundance and fold-change (*Polg*/WT) as shown in Fig. 5G and Fig. S4K

Provided as a separate file (.xlsx) due to size.

Table S6.

m/z and retention time for LC/MS (iHILIC) analysis

Metabolite	Formula	m/z (negative mode)	Retention time (min)
3-Hydroxybutyric acid	C4H8O3	103.0401	2.9
Acetoacetate	C4H6O3	101.0244	2.6
Adenine	C5H5N5	134.0472	3.3
Adenosine	C10H13N5O4	266.0895	3.3
ADP	C10H15N5O10P2	426.0221	9.9
AMP	C10H14N5O7P	346.0558	8.3
ATP	C10H16N5O13P3	505.9885	10.9
GMP	C10H14N5O8P	362.0507	10.4
Guanosine	C10H13N5O5	282.0844	6.1
Hypoxanthine	C5H4N4O	135.0312	3.8
IMP	C10H13N4O8P	347.0398	9.6
Inosine	C10H12N4O5	267.0735	4.4
Uric Acid	C5H4N4O3	167.0211	6.1
Xanthine	C5H4N4O2	151.0261	4.6

Table S7.

Ion transitions for LC/MS (Accucore C30) analysis

Lipid Species	Precursor Ion	Product Ion
Triacylglycerols (TAG)		
TAG(48:0)	824.8	551.5
TAG(48:3)	818.7	547.5
TAG(50:2)	848.8	549.5
TAG(50:3)	846.8	549.5
TAG(52:1)	878.8	605.6
TAG(52:2)	876.8	577.5
Diacylglycerols (DAG)		
DAG(16:0/18:1)	612.6	313.3
DAG(16:0/18:2)	610.5	313.3
DAG(16:0/18:3)	608.5	313.3
DAG(16:0/22:6)	658.5	313.3
DAG(16:1/18:1)	610.5	311.0
Phosphatidylethanolamine (PE)		
PE(34:2)	716.5	575.5
PE(36:3)	742.5	601.5
PE(36:2)	744.6	603.5
PE(38:7)	762.5	621.5
PE(38:6)	764.5	623.5
PE(38:3)	770.6	629.6
PE(40:7)	790.5	649.5
Phosphatidylcholine (PC)		
PC(33:1)	746.6	184.1
PC(34:0)	762.6	184.1
PC(34:1)	760.6	184.1
PC(36:0)	790.6	184.1
PC(36:1)	788.6	184.1
PC(36:2)	786.6	184.1
PC(38:1)	816.6	184.1
PC(38:6)	806.6	184.1

Table S8.

Sequence for gene specific primers for quantitative polymerase chain reaction

Mouse gene	Forward primer	Reverse primer
Actb	GAGGTATCCTGACCCTGAAGTA	CACACGCAGCTCATTGTAGA
Pcx	AATGTCCGGCGTCTGGAGTA	ACGCACGAAACACTCGGAT
Pck1	CTGCATAACGGTCTGGACTTC	GCCTTCCACGAACTTCCTCAC
Pck2	ATGGCTGCTATGTACCTCCC	GCGCCACAAAGTCTCGAAC
Ampd2	CCTCTCCGCTACAGTCTGC	CTCCTTGCATTTGCCATCCAT
Ampd3	ATCCGGTCGCAGTCTTTGTC	CTCAGGCATAGCATAGGGTGC
Xdh	ATGACGAGGACAACGGTAGAT	TCATACTTGGAGATCATCACGGT
